# Lactylation-Boosted m^5^C RNA Modification Drives Choroidal Neovascularization

**DOI:** 10.34133/research.0913

**Published:** 2025-10-02

**Authors:** Sipeng Zuo, Lin Li, Jieling Tang, Fuxiang Ye, Sok, Yan Liu, Shengfang Ge, Renbing Jia, Xianqun Fan, Peiwei Chai, Linna Lu

**Affiliations:** ^1^ State Key Laboratory of Eye Health, Department of Ophthalmology, Shanghai Ninth People's Hospital, Shanghai Jiao Tong University School of Medicine, Shanghai, People’s Republic of China.; ^2^ Shanghai Key Laboratory of Orbital Diseases and Ocular Oncology, Shanghai, People’s Republic of China.; ^3^ Department of Ophthalmology, Shanghai General Hospital, Shanghai Jiao Tong University School of Medicine, Shanghai, People’s Republic of China.

## Abstract

As a prevalent posttranscriptional RNA modification, N5-methylcytosine (m^5^C) plays a crucial role in a multitude of cellular responses and processes that govern RNA metabolism. Howbeit, a comprehensive understanding of the role and mechanism of m^5^C and the methylase NSUN2 in choroidal neovascularization (CNV) remains elusive. Herein, we revealed that histone lactylation fuels NSUN2-mediated m^5^C modification, leading to up-regulated m^5^C levels and initiates the pathological progression of CNV. First, we discovered that NSUN2 expression and m^5^C modification level were markedly higher in CNV-endothelial cells (ECs) than in normal ECs, which was driven by the augmentation of lactate-mediated histone lactylation within the NSUN2 promoter. NSUN2 silencing inhibited proliferative, migration, and tube formation activities of ECs. Importantly, compared with EC *Nsun2*^flox/flox^ mice, EC-specific *Nsun2*-deficient (EC *Nsun2*^−/−^) mice displayed less retinal vascular leakage after laser induction. Through multiomics analyses, we identified that NSUN2 enhanced m^5^C level of A-kinase anchoring protein 2 (AKAP2), activating protein kinase A (PKA)–vascular endothelial growth factor receptor 2 (VEGFR2) signaling in ECs. Overall, our findings revealed that the interplay between histone lactylation and m^5^C RNA modification serves as an important pathological trigger for CNV, paving new avenues for the treatment of CNV.

## Introduction

In mammals, RNA molecules are frequently subjected to methylation, a common form of posttranscriptional modification [1]. N5-methylcytosine (m^5^C), which involves the methylation of the fifth carbon atom of the RNA cytosine base, stands out as a crucial modification pattern found across various types of RNA, including transfer RNAs (tRNAs), ribosomal RNAs (rRNAs), messenger RNAs (mRNAs), and mitochondrial RNAs [2]. DNA methyltransferase 2 (DNMT2) and members of the NSUN family (especially NSUN2) of methyltransferases are recognized as the key enzymes responsible for introducing m^5^C methylation in these RNA types [3–5].

Compelling evidence has emerged that highlights the profound impact of the dynamic m^5^C modification on a plethora of cellular processes. These include the modulation of RNA structure, stability, and translation efficiency [6]. For example, a regulatory feedback loop involving NSUN2 and E2F transcription factor 1 (E2F1), modulated by m^5^C, has been shown to drive tumor progression in ovarian cancer [7]. Additionally, NSUN2, when activated by glucose, directly stabilizes three prime repair exonuclease 2 (TREX2), a factor that fosters tumorigenesis and confers resistance to anti-programmed death ligand 1 (PD-L1) immunotherapy [8]. Moreover, the role of NSUN2 in mediating m^5^C methylation has been implicated in impairing endometrial receptivity, leading to recurrent implantation failure in reproductive health [9].

Choroidal neovascularization (CNV) is a pathological process characterized by the formation of new, aberrant blood vessels originating from the choroid [10]. This condition is particularly susceptible to rapid, malignant progression, often observed in diseases such as neovascular age-related macular degeneration (nAMD) and pathological myopia [10]. In its advanced stages, CNV can lead to permanent vision loss. A multitude of factors have been implicated in CNV development, encompassing hypoxia, inflammatory reactions, and alterations in the extracellular matrix [11]. Importantly, recent research has shed light on the connection between RNA methylation and vascularization processes. For instance, methyltransferase-like 3 (METTL3)-dependent N6-methyladenosine mRNA modification promoted angiogenesis in the retinopathy model [12,13]. Additionally, N6-methyladenosine demethylation mediated by fat mass- and obesity-associated protein (FTO) could regulate pathological angiogenesis during CNV [14]. Despite these insights, the intricate mechanisms by which m^5^C RNA methylation contributes to the pathogenesis of CNV remain largely obscure.

Endothelial cells (ECs) play a critical role in angiogenesis within pathological tissues, serving as key regulators of vascular formation and remodeling in disease contexts [15–17]. Emerging evidence have proved that ECs are highly dependent on glycolysis to produce more than 80% adenosine triphosphate (ATP) for their own activities, which may facilitate EC retention and delivery of oxygen to surrounding tissues [16]. These conclusions are consistent with the results that glycolysis blockade leads to inhibition of vascularization [18,19]. However, the relationship between lactate, a byproduct of glycolysis, and the biological functions of ECs remains enigmatic. Recently, lactate-related metabolic and epigenetic reprogramming processes have been established as crucial players in a multitude of cellular pathological mechanisms [20,21]. Specifically, lactylation-driven METTL3-mediated RNA m^6^A modification promotes immunosuppression of tumor-infiltrating myeloid cells [22]. In the ocular diseases, histone lactylation-boosted AlkB homolog 3 (ALKBH3) potentiates ocular melanoma progression [23]. These results indicated that histone lactylation induced by lactic acid derived from glycolysis, including H3K18la, promoted pathological angiogenesis during CNV. H3K18la enhanced the transcription of multiple downstream target genes, which reinforced the mechanistic link between H3K18la and NSUN2 transcription [24].

In the present study, we elucidated the pathological role of NSUN2-dependent m^5^C modification in CNV progression. Our results suggested that NSUN2 up-regulation due to histone lactylation increased m^5^C modification levels in CNV. Moreover, compared with EC *Nsun2*^flox/flox^ mice, EC-specific *Nsun2*-deficient (EC *Nsun2*^−/−^) mice displayed less retinal vascular leakage after laser induction. Mechanistically, NSUN2-mediated m^5^C increased A-kinase anchoring protein 2 (AKAP2) mRNA stability and vascular endothelial growth factor receptor 2 (VEGFR2) expression to promote CNV formation. These data suggested that histone lactylation-driven NSUN2 hyperexpression is critical for CNV progression, thus providing a novel promising therapeutic strategy for CNV-related diseases.

## Results

### m^5^C and NSUN2 expression level is elevated in pathological vascular ECs

**Fig. 1.**
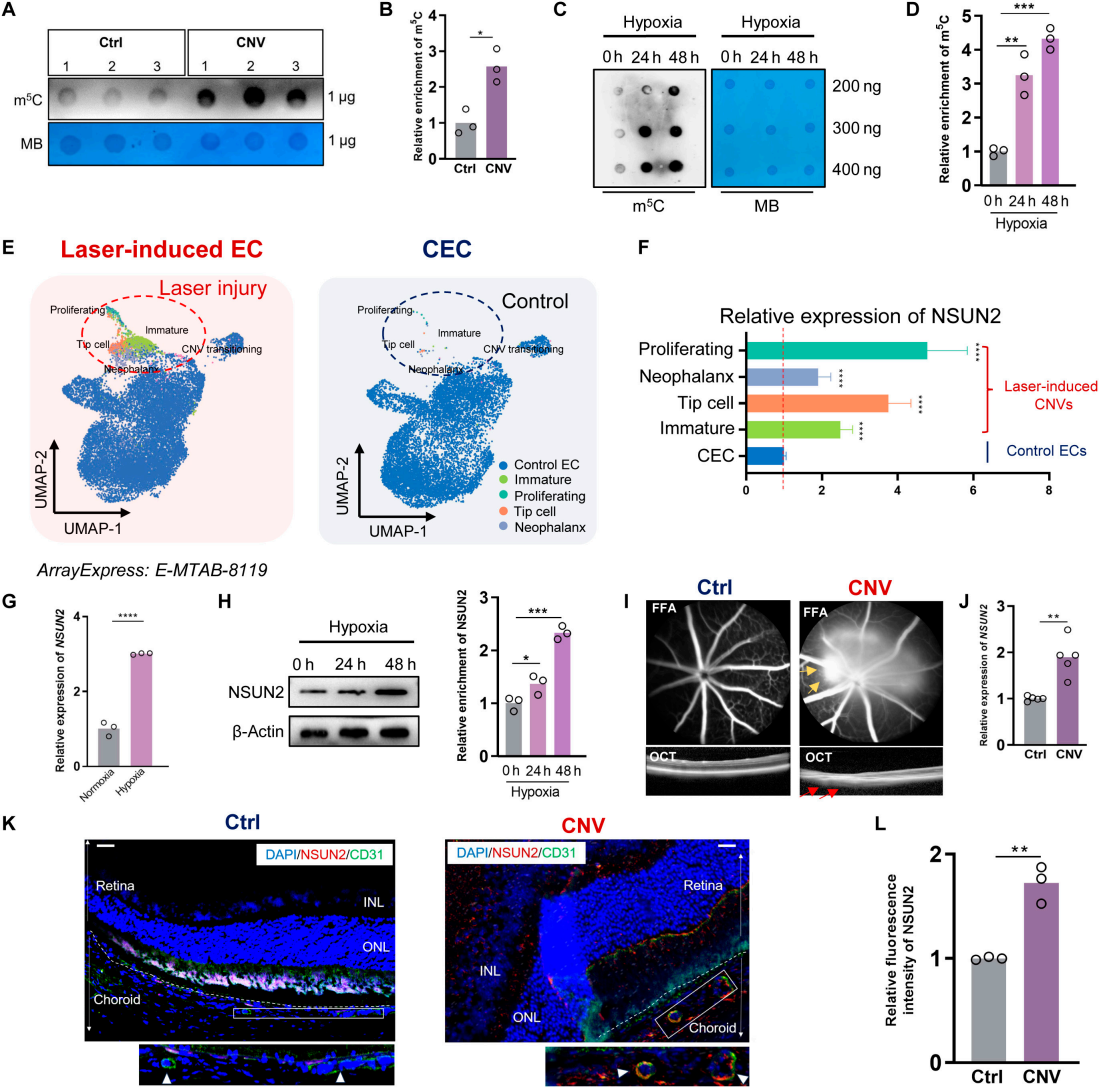
m^5^C and NSUN2 expression levels are elevated in pathological vascular endothelial cells (ECs). (A and B) Dot blot showing the m^5^C signal compared to the methylene blue signal in the retinal pigment epithelium (RPE)–choroid complex of control and choroidal neovascularization (CNV)-bearing mice (*n* = 3). The data are presented as the mean ± SD of experimental triplicates. Significance was determined by an unpaired 2-tailed Student’s *t* test. *P < 0.05. (C and D) Dot blot showing the m^5^C signal compared to the methylene blue signal in hypoxic human umbilical vein endothelial cells (HUVECs) (*n* = 3). The data are presented as the mean ± SD of experimental triplicates. Significance was determined by an unpaired 2-tailed Student’s *t* test. *P < 0.05. (E) Uniform Manifold Approximation and Projection (UMAP) representation of single cells from CNV-ECs and healthy ECs labeled with cell types under ArrayExpress: E-MTAB-8119. (F) Histogram showing expression level of NSUN2 in different CNV-EC clusters. (G) Quantitative polymerase chain reaction (qPCR) data showing NSUN2 expression in hypoxic and control HUVECs (*n* = 3). The data are presented as the mean ± SD of experimental replication. Significance was determined by an unpaired 2-tailed Student’s *t* test. ****P < 0.0001. (H) Western blot (WB) data showing NSUN2 expression in hypoxic and control HUVECs (*n* = 3). The data are presented as the mean ± SD of experimental triplicates. Significance was determined by an unpaired 2-tailed Student’s *t* test. **P < 0.01, ***P < 0.001. (I) Fundus fluorescein angiography (FFA) and OCT images in control and CNV-bearing mice. (J) qPCR data showing NSUN2 expression in the RPE–choroid complex of control and CNV-bearing mice (*n* = 5). The data are presented as the mean ± SD of experimental replication. Significance was determined by an unpaired 2-tailed Student’s *t* test. **P < 0.01. (K and L) Immunofluorescence of NSUN2 (red), CD31 (green), and 4′,6-diamidino-2-phenylindole (DAPI) staining (blue) and statistical analysis in control and CNV-bearing mice (*n* = 3). Scale bars, 20 μm. The data are presented as the mean ± SD of experimental triplicates. Significance was determined by an unpaired 2-tailed Student’s *t* test. **P < 0.01.

Firstly, we compared the levels of m^5^C modification on the retinal pigment epithelium (RPE)/choroid complex between CNV-bearing mice and control mice in order to investigate the potential effects of m^5^C modification on CNV pathogenesis. Resulting from an anti-m^5^C dot blot assay, the choroidal endothelial cells (CECs) from CNV-bearing mice were found to contain elevated levels of m^5^C modification (Fig. 1A and B). Given the fact that hypoxia plays a pivotal role in ocular angiogenesis [25], we consistently detected elevated m^5^C modification in human umbilical vein endothelial cells (HUVECs) along with its exposure to hypoxia (Fig. 1C and D).

A separate population of CECs in lasered mice, representing CNV-ECs (immature, proliferating, tip cell, neophalanx), has been identified in previous single-cell analysis [26] (Fig. 1E, ArrayExpress: E-MTAB-8119). Differential gene analysis showed that in CNV-ECs, the m^5^C methyltransferase NSUN2 exhibits a robust higher expression level compared to control ECs (Fig. 1F). Importantly, the RNA and protein expression of NSUN2 was up-regulated in both the hypoxic HUVECs (Fig. 1G and H) and CNV mouse model (Fig. 1I to L) when compared to the control group. In conclusion, these observations suggest that the overexpression of m^5^C methyltransferase NSUN2 potentially contributes to the development of CNV.

### NSUN2 promotes proliferation, migration, tube formation, and angiogenesis of ECs

**Fig. 2.**
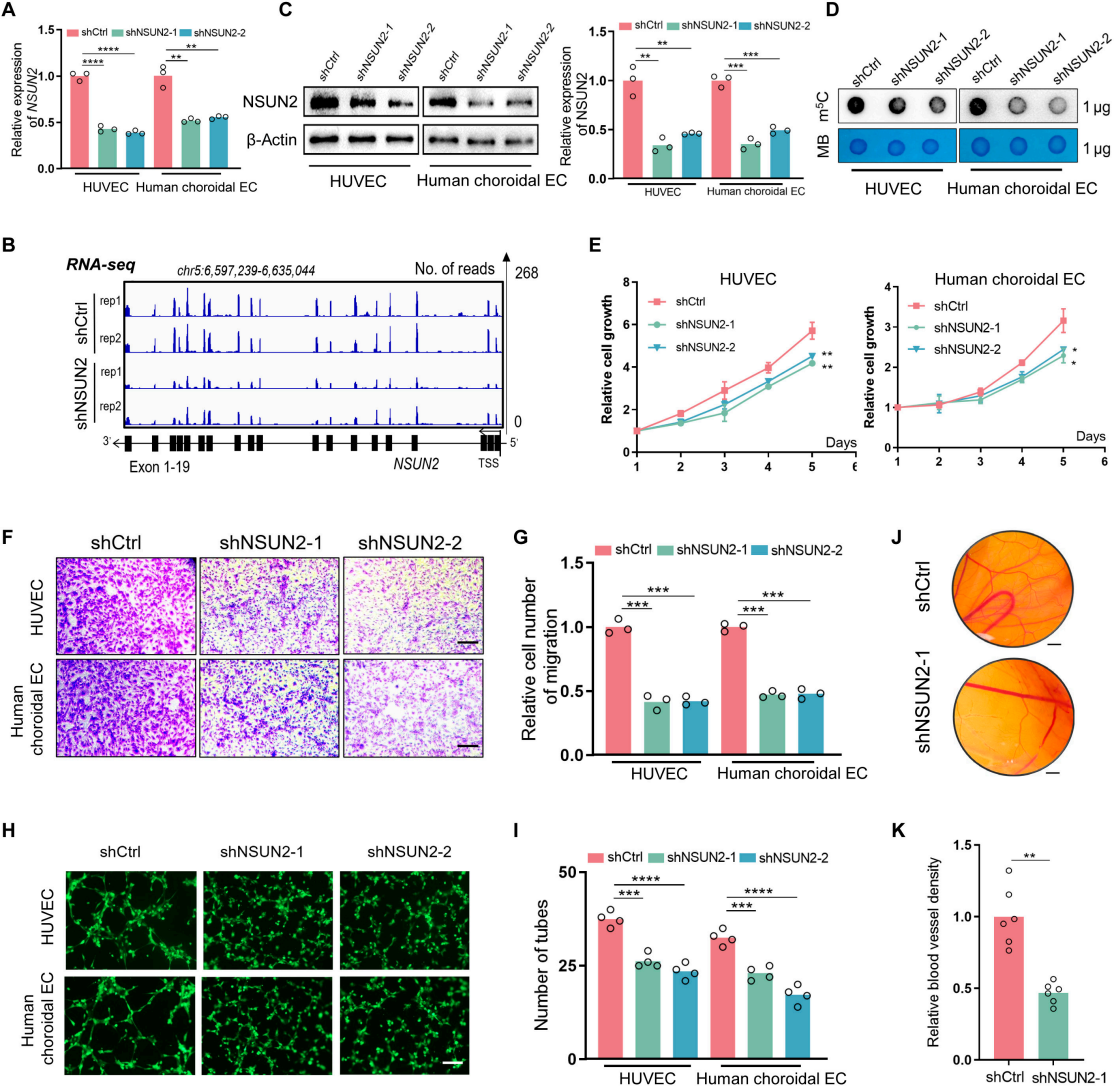
NSUN2 promotes proliferation, migration, tube formation, and angiogenesis of ECs. (A) qPCR data showing NSUN2 expression in ECs following NSUN2 knockdown (*n* = 3). The data are presented as the mean ± SD of experimental triplicates. Significance was determined by an unpaired 2-tailed Student’s *t* test. **P < 0.01, ****P < 0.0001. (B) Integrative Genomics Viewer (IGV) showing the expression of NSUN2 in HUVECs following NSUN2 knockdown. (C) WB showing NSUN2 expression in ECs following NSUN2 knockdown (*n* = 3). The data are presented as the mean ± SD of experimental triplicates. Significance was determined by an unpaired 2-tailed Student’s *t* test. **P < 0.01, ***P < 0.001. (D) Dot blot showing the m^5^C signal relative to the methylene blue signal in ECs following NSUN2 knockdown (*n* = 3). (E) A Cell Counting Kit-8 (CCK-8) assay was employed to evaluate the proliferation of ECs after NSUN2 knockdown (*n* = 3). The data are presented as the mean ± SD of experimental triplicates. Significance was determined by an unpaired 2-tailed Student’s *t* test. *P < 0.05, **P < 0.01. (F and G) A transwell assay was employed to evaluate the migration of ECs following NSUN2 knockdown (*n* = 3). Scale bars, 100 μm. Representative images from 3 experimental replicates are shown. The data are presented as the mean ± SD. Significance was determined by an unpaired 2-tailed Student’s *t* test. ***P < 0.001. (H and I) A tube formation assay was employed to evaluate the tube formation of ECs following NSUN2 knockdown (*n* = 4). Scale bars, 100 μm. Representative images from 3 experimental replicates are shown. The data are presented as the mean ± SD. Significance was determined by an unpaired 2-tailed Student’s *t* test. ***P < 0.001, ****P < 0.0001. (J and K) Representative images from experimental replication and quantitative analysis of blood vessels of chorioallantoic membrane (CAM) treated with conditional medium from NSUN2-silenced cells and control cells (*n* = 5). Scale bars, 500 μm. Representative images from 3 experimental replicates are shown. The data are presented as the mean ± SD. Significance was determined by an unpaired 2-tailed Student’s *t* test. **P < 0.01.

To investigate the role of NSUN2 in abnormal progression of CNV, we generated 2 individual short hairpin RNAs (shRNAs) for silencing NSUN2, in both HUVECs and primary CECs (Table S1). After shRNA transfection, NSUN2 expression was diminished in both cells, as demonstrated by quantitative reverse transcription polymerase chain reaction (qRT-PCR) (Fig. 2A), RNA-sequencing (RNA-seq) [for HUVEC, deposited in Gene Expression Omnibus (GEO) database: GSE267662; Fig. 2B], and Western blot (WB; Fig. 2C) assays. In addition, dot blot assay indicated decreased levels of m^5^C following the silencing of NSUN2 in 2 ECs (Fig. 2D). The composition of CNV-ECs has been demonstrated to encompass 2 beforehand cell types, namely, “CNV transitioning” and “immature” cells, along with 3 advanced cell types: “tip cell”, “proliferating cell”, and “neophalanx” [26]. As revealed by gene ontology (GO) enrichment analysis, CNV-ECs heavily rely on their intrinsic abilities in proliferation and migration to fulfill the demands of vascular remodeling (Fig. S1A to E). Therefore, we detected proliferation, migration, tube formation, and angiogenesis abilities of ECs. As a result, a remarkable decrease in proliferation rate was observed compared to the vector group (Fig. 2E). Additionally, NSUN2 silencing impeded the migration of ECs (Fig. 2F and G). Furthermore, knockdown of NSUN2 also compromised the tube formation capacity of ECs in vitro (Fig. 2H and I). In addition, we performed a chorioallantoic membrane (CAM) assay using the conditioned medium derived from HUVECs to evaluate the role of NSUN2 in angiogenesis in vivo. Interestingly, ECs silenced for NSUN2 showed decreased proangiogenic capacity (Fig. 2J and K). Quantitative PCR confirmed that NSUN2 loss reduced the mRNA levels of key pro-angiogenic genes—*ITGAV*, *ITGB3*, *FGF2*, and *ANGPT2* (Fig. S2A to D)—all of which are known to be essential for endothelial sprouting and vessel stabilization [11,27,28]. Thus, conditioned medium from NSUN2-deficient ECs contains lower concentrations of these pro-angiogenic factors and, when applied to the CAM, results in a measurable reduction in neovascularization. This approach directly links NSUN2-dependent endothelial secretome changes to functional angiogenesis in vivo, providing mechanistic insight that could not be obtained from the CAM assay alone. Taken together, these data indicated that NSUN2 could promote angiogenesis of ECs both in vitro and in vivo.

### NSUN2 facilitates the progression of laser-induced CNV and retinal vascular leakage

**Fig. 3.**
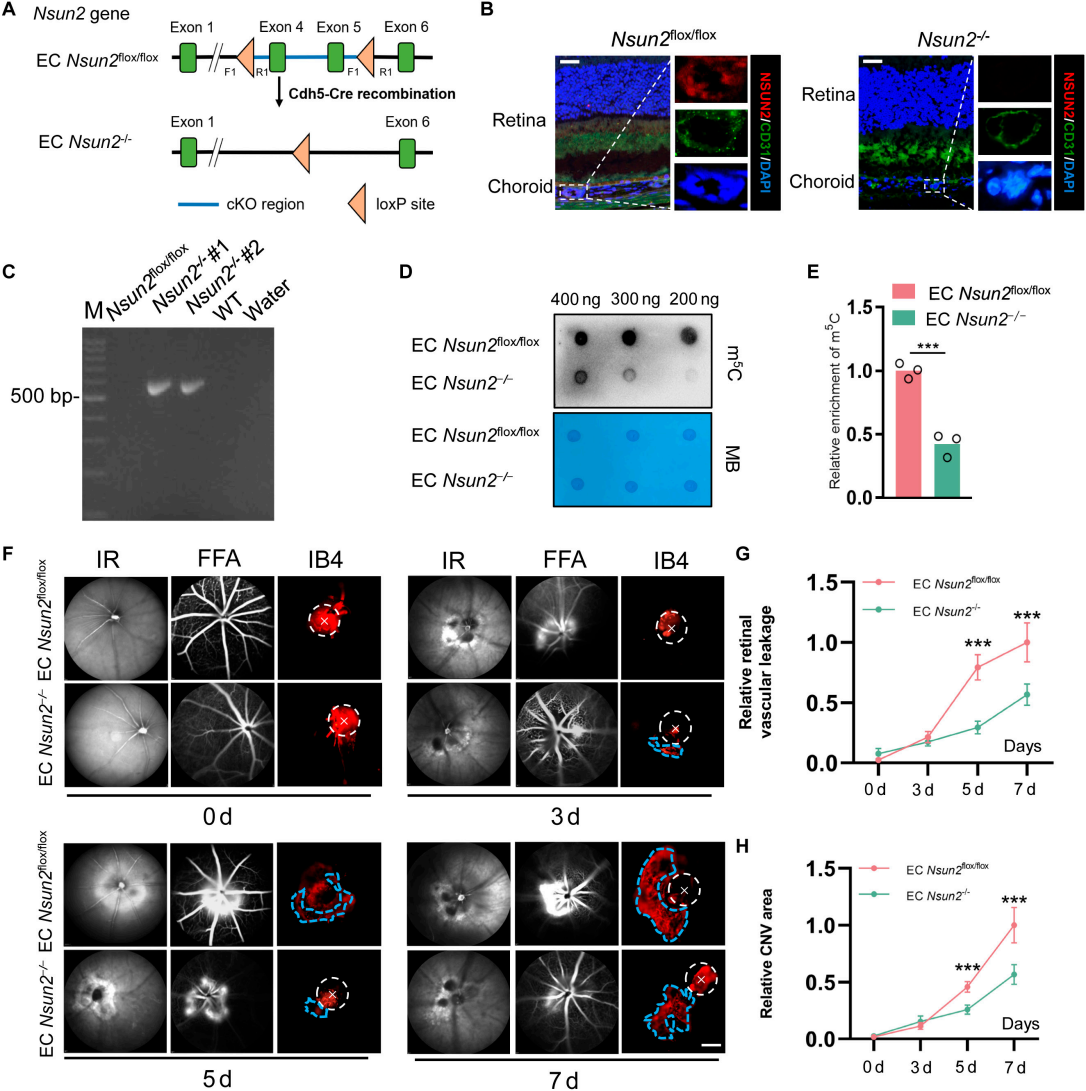
NSUN2 facilitates the progression of laser-induced CNV and retinal vascular leakage. (A) Schematic diagram showing the generation of EC-specific *Nsun2*-deficient mice. (B) Immunofluorescence of NSUN2 (red), CD31 (green), and DAPI staining (blue) in EC *Nsun2*^flox/flox^ and EC *Nsun2*^−/−^ mice (*n* = 3). Scale bars, 20 μm. (C) PCR genotyping verified *Nsun2* exon 4, 5 deletions in EC *Nsun2*^−/−^ mice (*n* = 3). (D and E) Dot blot and statistical analysis showing the m^5^C signal relative to the methylene blue signal in EC *Nsun2*^flox/flox^ and EC *Nsun2*^−/−^ mice (*n* = 3). The data are presented as the mean ± SD of experimental triplicates. Significance was determined by an unpaired 2-tailed Student’s *t* test. ***P < 0.001. (F) Images of IR, FFA, and immunofluorescence in EC *Nsun2*^flox/flox^ and EC *Nsun2*^−/−^ mice after laser-induced CNV (*n* = 6). (G and H) Statistical analysis of retinal vascular leakage and CNV area in EC *Nsun2*^flox/flox^ and EC *Nsun2*^−/−^ mice after laser-induced CNV (*n* = 5). The data are presented as the mean ± SD of experimental replication. Significance was determined by an unpaired 2-tailed Student’s *t* test. ***P < 0.001.

To investigate the role of NSUN2 in CNV, we generated EC-specific *Nsun2*-deficient mice, as depicted in Fig. 3A. Immunofluorescence staining of retinal vasculature in mice confirmed the absence of NSUN2 protein in ECs (Fig. 3B). The successful cre-mediated recombination of the *Nsun2* locus in EC *Nsun2*^−/−^ mice was confirmed through PCR analysis of genomic DNA extracted from primary mouse retinal ECs [~593 base pairs (bp); Fig. 3C]. Concurrently, the Sanger sequencing result revealed the absence of *Nsun2* gene fragment (Fig. S3A and B). We extracted and isolated mRNA from EC *Nsun2*^−/−^ mice, and dot blot assay revealed reduced levels of m^5^C in EC *Nsun2*^−/−^ mice (Fig. 3D and E). Moreover, laser-induced CNV mouse model was established and the infrared spectroscopy (IR) fundus images showed laser spot position around the optic disc (Fig. 3F, left). Besides, fundus fluorescein angiography (FFA) revealed that endothelial depletion of NSUN2 alleviated observably laser-induced retinal vascular leakage (Fig. 3F, middle, and G). Consistent with this, EC *Nsun2*^−/−^ mice exhibited a significant 69.4% reduction in the area of CNV lesions (Fig. 3F, right, and H). Consequently, these results indicate NSUN2 is in charge of the progression of CNV and retinal vascular leakage.

### The identification of AKAP2 as a potential NSUN2-mediated target

**Fig. 4.**
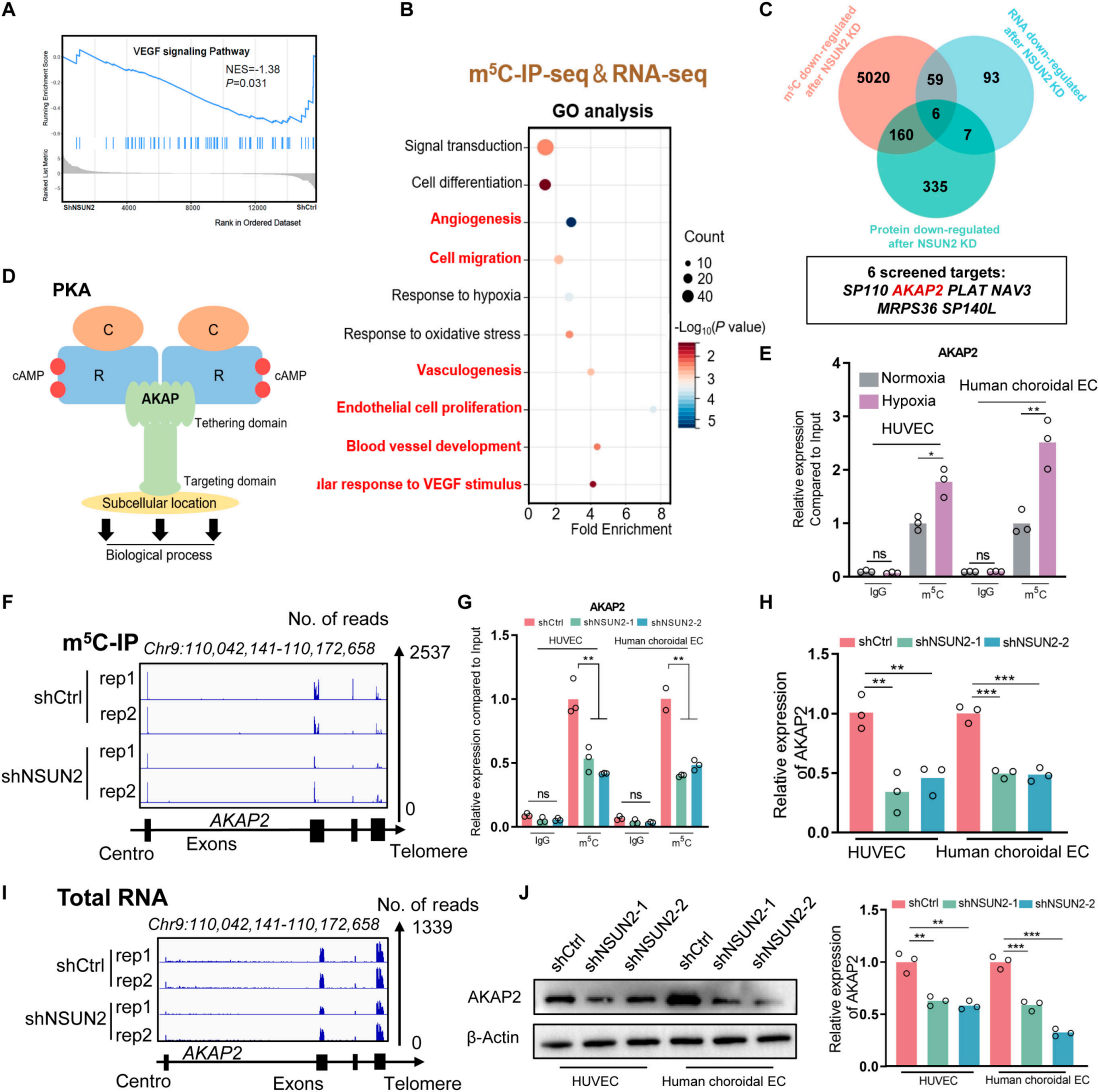
The identification of A-kinase anchoring protein 2 (AKAP2) as a potential NSUN2-mediated target. (A) GSEA analysis showing the VEGF signaling pathway following NSUN2 knockdown in HUVECs. (B) GO analysis of both RNA expression and m^5^C modification down-regulated genes in HUVECs. (C) Multiomics analysis identified downstream targets of NSUN2. (D) Schematic diagram showing the role of AKAP2. (E) m^5^C-meRIP-qPCR assay of m^5^C status in AKAP2 in HUVECs under hypoxia treatment (*n* = 3). The data are presented as the mean ± SD of experimental triplicates. Significance was determined by an unpaired 2-tailed Student’s *t* test. *P < 0.05, **P < 0.01, ns indicates no significance. (F) IGV tracks from m^5^C-meRIP-seq analysis showing m^5^C enrichment of AKAP2. (G) m^5^C-MeRIP-qPCR assay of m^5^C status in AKAP2 in HUVECs following NSUN2 knockdown (*n* = 3). The data are presented as the mean ± SD of experimental triplicates. Significance was determined by an unpaired 2-tailed Student’s *t* test. **P < 0.01. (H) qPCR assay showing AKAP2 expression in ECs following NSUN2 knockdown (*n* = 3). The data are presented as the mean ± SD of experimental triplicates. Significance was determined by an unpaired 2-tailed Student’s *t* test. **P < 0.01, ***P < 0.001. (I) IGV tracks from RNA-sequencing (RNA-seq) analysis showing expression of AKAP2. (J) WB data showing AKAP2 expression in ECs following NSUN2 knockdown (*n* = 3). The data are presented as the mean ± SD of experimental triplicates. Significance was determined by an unpaired 2-tailed Student’s *t* test. **P < 0.01, ***P < 0.001.

To elucidate the underlying mechanism by which NSUN2 facilitates the pathogenesis of CNV, we conducted comprehensive multiomics analyses to identify potential RNA targets of NSUN2, encompassing transcriptome analysis (RNA-seq, deposited in GSE267662, differentially expressed genes are defined as |FC| > 1.5 or |FC| < 0.67, *P* < 0.01), proteomic analysis (iTRAQ, Table S3, log_2_|FC| > 0.5 or |FC| < −0.5, *P* < 0.05), and m^5^C-immunoprecipitation sequencing (IP-seq) (deposited in GEO database: GSE267662, differentially expressed genes are defined as log_2_|FC| > 6.0, *P* < 0.05). Gene Set Enrichment Analysis (GSEA) indicated that the VEGF signaling pathway was markedly down-regulated in ECs upon NSUN2 knockdown (Fig. 4A; according to RNA-seq, NES = −1.38, *P* = 0.031). Importantly, the altered genes with expression level as well as m^5^C modification down-regulation after NSUN2 silencing are highly related to several pivotal events in ECs, including angiogenesis, cell migration, cell proliferation, and cellular response to VEGF stimulus (Fig. 4B). To identify the functional target of NSUN2 in CNV, a detailed evaluation of aforementioned multiomics was conducted and 6 potential targets were selected for further consideration (Fig. 4C).

A comprehensive comparison of the 6 convergent candidates (*AKAP2*, *SP110*, *PLAT*, *NAV3*, *MRPS36*, and *SP140L*) is provided in Table S4. AKAP2 was selected for mechanistic follow-up because it is one of highest m^5^C stoichiometry reduction (meRIP-seq log₂FC = −6.06, *P* = 9.024 × 10^−8^), one of largest transcript-level down-regulation (RNA-seq log₂FC = −0.71, *P* = −0.005), and one of most pronounced protein-level decrease (iTRAQ log₂FC = −0.53, *P* = 0.007). In addition, the endothelial functions of PLAT (primarily fibrinolysis), NAV3 (neuronal guidance), and the remaining candidates either are less relevant to sprouting angiogenesis or have been less well characterized in ECs, whereas AKAP2 is an established scaffold protein that integrates protein kinase A (PKA) and Src signaling to control endothelial migration and tube formation [29,30].

*AKAP2*, encoding a scaffolding protein that isolates PKA to specific subcellular locations by binding to its regulatory subunit (Fig. 4D), was down-regulated at the RNA level (Fig. 4C, blue box; RNA-seq: shNSUN2 versus shCtrl, |FC| = 0.61, *P* < 0.01), m^5^C modification level (Fig. 4C, orange box; m^5^C-meRIP-seq: shNSUN2 versus shCtrl, log_2_|FC| = −6.058, *P* < 0.05), and protein level (Fig. 4C, green box; iTRAQ: shNSUN2 versus shCtrl, log_2_|FC| = −0.53, *P* < 0.05) in NSUN2-deficient ECs. In addition, m^5^C-methylated RNA immunoprecipitation (meRIP)-PCR indicated that AKAP2 was hypermethylated in ECs exposed to hypoxia (Fig. 4E). Interestingly, decreased m^5^C modifications of AKAP2 mRNA upon NSUN2 silencing were identified by m^5^C-meRIP-seq (Fig. 4F) and m^5^C-meRIP-PCR (Fig. 4G), following a remarkable down-regulation of *AKAP2* RNA (Fig. 4H and I) and protein (Fig. 4J) levels.

### The pro-angiogenesis of NSUN2 depends on AKAP2 activation

The findings of previous studies have demonstrated that AKAP2 forms a signaling complex with PKA and steroid receptor coactivator 3 (Src3), thereby facilitating the up-regulation of VEGFA expression [30]. Subsequently, we found that both *AKAP2* RNA (Fig. S4A) and protein (Fig. S4B) levels were elevated in the hypoxic ECs. Also, the CNV mouse model exhibited a significant increase in AKAP2 expression, in both RNA (Fig. S4C) and protein levels (Fig. S4D).

**Fig. 5.**
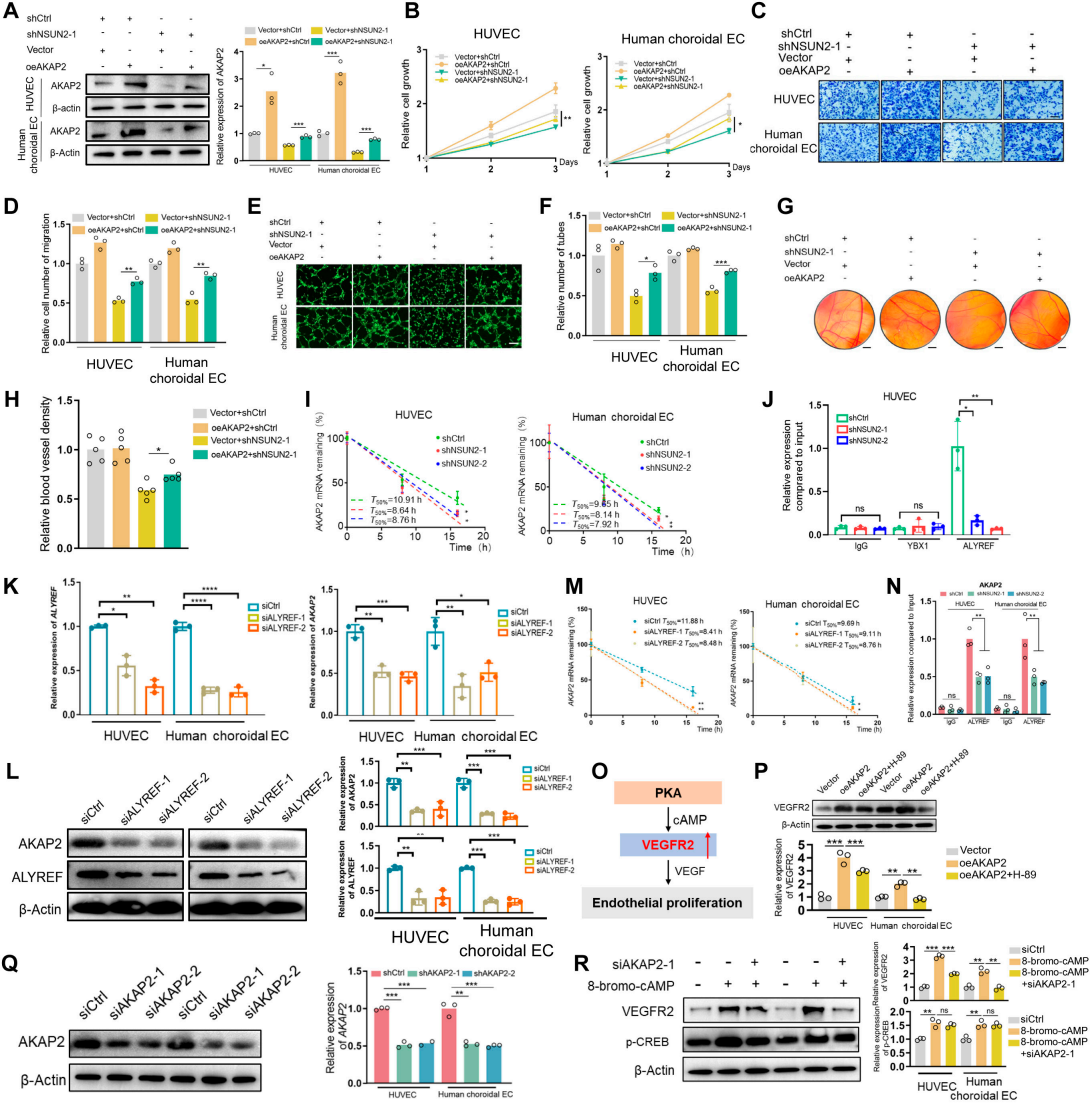
The pro-angiogenesis of NSUN2 depends on AKAP2 activation. (A) WB data showing AKAP2 expression in ECs following NSUN2 knockdown and AKAP2 overexpression (*n* = 3). The data are presented as the mean ± SD of experimental triplicates. Significance was determined by an unpaired 2-tailed Student’s *t* test. **P < 0.01, ***P < 0.001. (B) A CCK-8 assay was performed to assess the proliferation of NSUN2-deficient ECs after AKAP2 overexpression (*n* = 3). The data are presented as the mean ± SD of experimental triplicates. Significance was determined by an unpaired 2-tailed Student’s *t* test. *P < 0.05, **P < 0.01. (C and D) A transwell assay was employed to evaluate the migration of NSUN2-deficient ECs after AKAP2 overexpression (*n* = 3). Scale bars, 100 μm. Representative images from 3 experimental replicates are shown. The data are presented as the mean ± SD. Significance was determined by an unpaired 2-tailed Student’s *t* test. **P < 0.01. (E and F) A tube formation assay was employed to evaluate the tube formation of NSUN2-deficient ECs after AKAP2 overexpression (*n* = 3). Scale bars, 100 μm. Representative images from 3 experimental replicates are shown. The data are presented as the mean ± SD. Significance was determined by an unpaired 2-tailed Student’s *t* test. *P < 0.05, ***P < 0.001. (G and H) Representative images from experimental replication and quantitative analysis of blood vessels of CAM treated with conditional medium from NSUN2-deficient ECs after AKAP2 overexpression (*n* = 5). Scale bars, 500 μm. Representative images from 3 experimental replicates are shown. The data are presented as the mean ± SD. Significance was determined by an unpaired 2-tailed Student’s *t* test. *P < 0.05. (I) Half-life of AKAP2 in NSUN2-deficient ECs treated with actinomycin (10 μg/ml) for 0 to 16 h (*n* = 3). *P < 0.05, **P < 0.01. (J) RIP-qPCR assay of AKAP2 expression in HUVECs induced by ALYREF and YBX1 (*n* = 3). The data are presented as the mean ± SD of experimental triplicates. Significance was determined by an unpaired 2-tailed Student’s *t* test. *P < 0.05, **P < 0.01. (K) qPCR data showing AKAP2 expression in ECs following ALYREF knockdown (*n* = 3). The data are presented as the mean ± SD of experimental triplicates. Significance was determined by an unpaired 2-tailed Student’s *t* test. *P < 0.05, **P < 0.01, ***P < 0.001, ****P < 0.0001. (L) WB data showing AKAP2 and ALYREF levels following ALYREF knockdown in ECs (*n* = 3). The data are presented as the mean ± SD of experimental triplicates. Significance was determined by an unpaired 2-tailed Student’s *t* test. **P < 0.01, ***P < 0.001. (M) Half-life of AKAP2 in ALYREF-deficient ECs treated with actinomycin (10 μg/ml) for 0 to 16 h (*n* = 3). *P < 0.05, **P < 0.01. (N) RIP-qPCR assay in AKAP2 following NSUN2 knockdown (*n* = 3). The data are presented as the mean ± SD of experimental triplicates. Significance was determined by an unpaired 2-tailed Student’s *t* test. **P < 0.01. (O) Schematic diagram showing the effect of PKA-VEGFR2 signaling transduction. (P) WB data showing VEGFR2 expression following AKAP2 overexpression with H89 in ECs (*n* = 3). The data are presented as the mean ± SD of experimental triplicates. Significance was determined by an unpaired 2-tailed Student’s *t* test. **P < 0.01, ***P < 0.001. (Q) WB data showing AKAP2 expression following AKAP2 knockdown in ECs (*n* = 3). The data are presented as the mean ± SD of experimental triplicates. Significance was determined by an unpaired 2-tailed Student’s *t* test. **P < 0.01, ***P < 0.001. (R) WB data showing VEGFR2 and p-CREB expression following AKAP2 knockdown with 8-bromo-cAMP (cyclic adenosine monophosphate) in ECs (*n* = 3). The data are presented as the mean ± SD of experimental triplicates. Significance was determined by an unpaired 2-tailed Student’s *t* test. **P < 0.01, ***P < 0.001.

To further confirm the role of AKAP2 in NSUN2-mediated pro-angiogenesis, we reintroduced AKAP2 into NSUN2-silenced ECs, in both HUVEC and primary CECs (Fig. 5A). Importantly, AKAP2 reintroduction compromised cell growth (Fig. 5B) and migration inhibition (Fig. 5C and D) in NSUN2-deficient ECs. Moreover, AKAP2 reintroduction remarkably resisted tube formation impairment in NSUN2-silenced ECs (Fig. 5E and F). Next, the CAM assay demonstrated that the restoration of AKAP2 rescued the pro-angiogenic capacity of NSUN2-silenced ECs (Fig. 5G and H). Collectively, these results provide further validation that AKAP2 acts as an indispensable downstream effector of NSUN2-mediated pro-angiogenesis, both in vivo and in vitro.

To test whether AKAP2 is required for NSUN2-driven angiogenesis, we performed an epistasis experiment in which NSUN2 was exogenously overexpressed in AKAP2-knockdown ECs (Fig. S5A). In control ECs, NSUN2 overexpression robustly enhanced proliferation (1.73-fold, *P* < 0.001) and trans-well migration (1.81-fold, *P* < 0.001) (Fig. S5B to D). By contrast, when AKAP2 was silenced, the same NSUN2 overexpression yielded only marginal improvements (proliferation 1.26-fold, *P* = 0.18; migration 1.14-fold, *P* = 0.15) (Fig. S5B to D). Consistently, tube formation assays revealed that AKAP2 depletion abolished NSUN2-mediated increases in branch point number and total tube length (Fig. S5E and F).These reciprocal loss- and gain-of-function data demonstrate that AKAP2 is indispensable for NSUN2-dependent pro-angiogenic signaling, thereby completing the mechanistic link between NSUN2-mediated m^5^C modification and endothelial angiogenesis.

### ALYREF recognizes m^5^C-methylated AKAP2 and promotes its RNA stability

We subsequently investigated the intricate mechanism underlying NSUN2-mediated regulation of AKAP2 through m^5^C modification. Considering the crucial roles of m^5^C modifications in maintaining RNA stability [31], we initially investigated the potential regulatory effect of NSUN2 on *AKAP2* RNA stability. Consequently, the *AKAP2* RNA stability was dramatically decreased in NSUN2-deficient ECs (Fig. 5I). Furthermore, to explore the “reader” protein responsible for the recognition of AKAP2 m^5^C methylation, we examined whether Y-box binding protein-1 (YBX1) and Aly/REF export factor (ALYREF) can potentially interact with the AKAP2 transcript. RNA immunoprecipitation analysis demonstrated the specific binding of ALYREF to AKAP2 mRNA, while weak interaction was observed with YBX1 (Fig. 5J). In addition, the silencing of ALYREF (Fig. 5K and L) resulted in a significant decrease in both mRNA (Fig. 5K) and protein (Fig. 5L) levels of AKAP2, accompanied by a pronounced reduction in RNA stability (Fig. 5M). To directly test whether NSUN2-mediated m^5^C is necessary for ALYREF binding to AKAP2 mRNA, we performed ALYREF RIP-qPCR in both control and NSUN2-knockdown primary CECs. Consistent with the proposed reader model of ALYREF, NSUN2 silencing reduced the enrichment of AKAP2 mRNA in ALYREF immunoprecipitates by ~50% in both HUVEC and primary choroidal cell models (Fig. 5N).

Furthermore, we conducted additional investigations to assess the functional role of ALYREF in angiogenesis. Remarkably, knockdown of ALYREF in vascular ECs exhibited a significant decrease in proliferation rate (Fig. S6A) and impaired tube formation capacity (Fig. S6B). Moreover, silencing of ALYREF also led to attenuated EC migration ability (Fig. S6C). These results support the conclusion that ALYREF serves as an important recognition regulator of the m^5^C-methylated AKAP2 transcript.

According to the current model of vessel sprouting, a leading tip cell migrates into avascular regions, while proliferating stalk cells elongate the emerging sprout. Upon fusion of 2 adjacent newly formed sprouts, ECs differentiate into quiescent phalanx cells [32]. Furthermore, tip cells and stalk cells maintain a dynamic equilibrium through reciprocal modulation of angiogenic signaling pathways, including VEGF and Notch pathways [33]. Significantly, the hypoxic microenvironment in nAMD stimulates VEGF secretion and endothelial vascularization [14]. Given that activation of the PKA pathway induces the formation of the VEGF–VEGFR2 protein complex and up-regulates VEGFR2 protein expression (Fig. 5O) [34], and considering the pivotal role of AKAP2 in mediating downstream target protein fate within the PKA pathway [30], we postulate that AKAP2 modulates VEGFR2 expression levels through the PKA pathway. Consistently, WB assay confirmed VEGFR2 up-regulation in ECs following AKAP2 exogenous overexpression. Besides, PKA inhibitor H89 blocked AKAP2-induced VEGFR2 up-regulation and tube formation in HUVECs (Fig. 5P and Fig. S7A and B).

Additionally, small interfering RNA (siRNA) was employed to suppress *AKAP2* RNA levels (Fig. 5Q), and the subsequent WB assay demonstrated that inhibition of AKAP2 impeded 8-bromo-cAMP (cyclic adenosine monophosphate)-induced PKA activation-mediated up-regulation of VEGFR2 (Fig. 5R). Taken together, these data indicate that the pro-angiogenesis of NSUN2 depends on AKAP2 activation in ECs.

### Lactate dehydrogenase A-mediated histone lactylation enhances the excessive expression of NSUN2

**Fig. 6.**
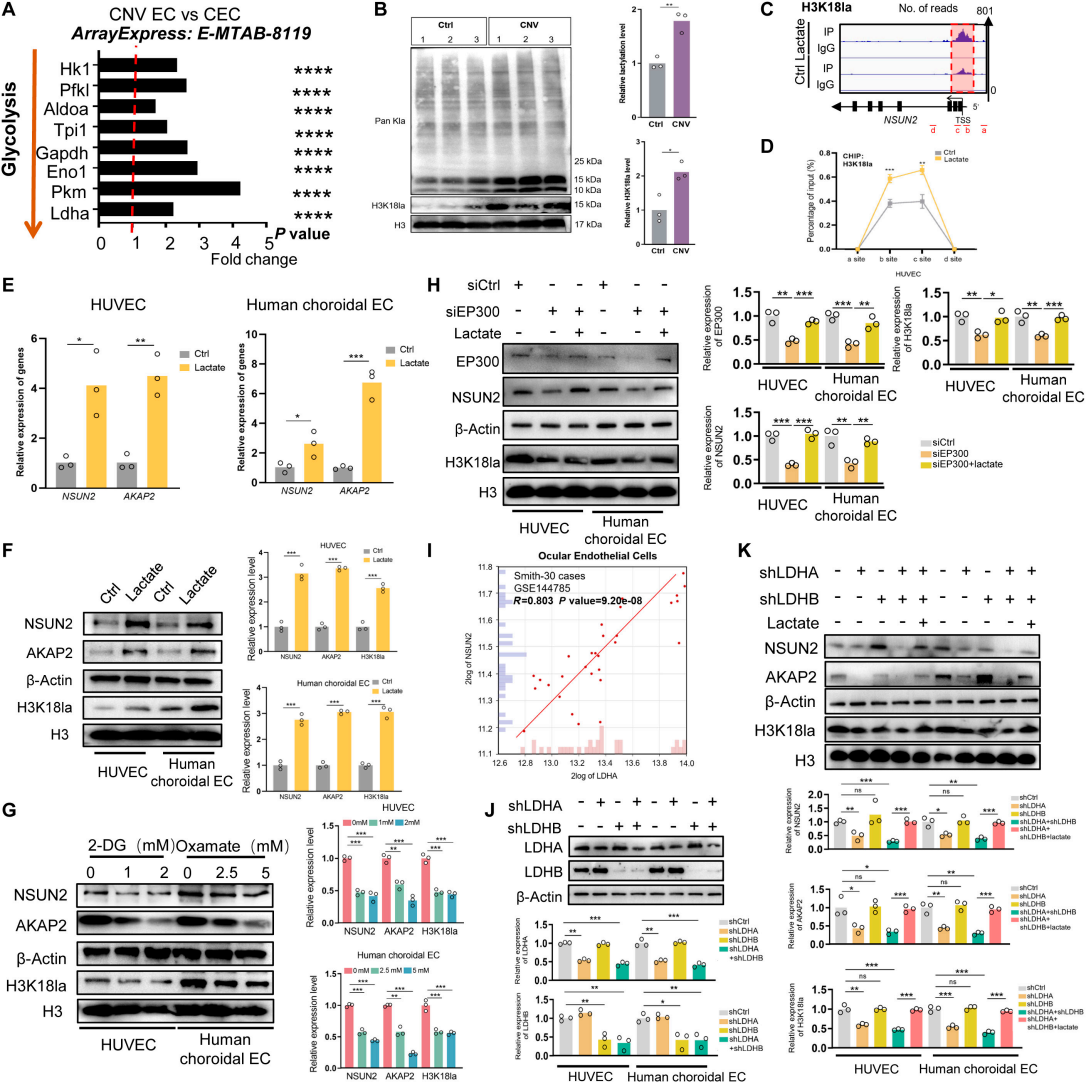
LDHA-mediated histone lactylation enhances the excessive expression of NSUN2. (A) GO analysis of up-regulated genes in CNV-ECs compared to control ECs. (B) WB data and statistical analysis showing Pan-Kla and H3K18la expression in the RPE–choroid complex of control and CNV-bearing mice (*n* = 3). The data are presented as the mean ± SD of experimental triplicates. Significance was determined by an unpaired 2-tailed Student’s *t* test. **P < 0.01, ***P < 0.001. (C) IGV tracks from CUT&Tag assay showing H3K18la enrichment of NSUN2. Sites a to d are distributed in the NSUN2 genomic region, and sites b and c are the H3K18la peaks. (D) CHIP-qPCR assay of H3K18la status in NSUN2 under lactate treatment (*n* = 3). The data are presented as the mean ± SD of experimental triplicates. Significance was determined by an unpaired 2-tailed Student’s *t* test. **P < 0.01, ***P < 0.001. (E and F) qPCR and WB assays showing NSUN2 and AKAP2 expression in ECs with 10 mM lactate (*n* = 3). The data are presented as the mean ± SD of experimental triplicates. Significance was determined by an unpaired 2-tailed Student’s *t* test. *P < 0.05, **P < 0.01, ***P < 0.001. (G) WB assays showing NSUN2, AKAP2, and H3K18la levels in ECs with 0, 1, and 2 mM 2-DG and 0, 2.5, and 5 mM oxamate (*n* = 3). The data are presented as the mean ± SD of experimental triplicates. Significance was determined by an unpaired 2-tailed Student’s *t* test. **P < 0.01, ***P < 0.001. (H) WB showing the expression level of EP300, NSUN2, and H3K18la. The data are presented as the mean ± SD of experimental triplicates. Significance was determined by an unpaired 2-tailed Student’s *t* test. *P < 0.05, **P < 0.01, ***P < 0.001. (I) GEO dataset showing the relevance between NSUN2 and LDHA. (J) WB assays showing LDHA and LDHB expression in ECs following LDHA and LDHB knockdown (*n* = 3). The data are presented as the mean ± SD of experimental triplicates. Significance was determined by an unpaired 2-tailed Student’s *t* test. **P < 0.01, ***P < 0.001. (K) WB assays showing NSUN2, AKAP2, and H3K18la levels in ECs (*n* = 3). The data are presented as the mean ± SD of experimental triplicates. Significance was determined by an unpaired 2-tailed Student’s *t* test. **P < 0.01, ***P < 0.001.

Due to the glycolytic addiction presented in ECs [16], the glycolytic genes were analyzed in CNV-ECs. The results revealed that the critical genes were up-regulated throughout the glycolytic pathway in CNV-ECs (Fig. 6A). Lactate, being a metabolite of glycolysis, prompted us to investigate the histone lactylation levels in CNV-bearing mice. Interestingly, there was a significant increase observed specifically around 15-kDa band within CNV-bearing mice, suggesting that the histone serves as the modified target of lactylation [17] (Fig. 6B). Further analysis confirmed significant enhancement specifically within CNV-bearing mice for H3K18la (Fig. 6B). To inspect the association between NSUN2 transcription and H3K18la, we initially conducted CUT&Tag assay, which revealed a substantial enrichment of H3K18la signal at the NSUN2 promoter region (Fig. 6C; deposited in GEO database: GSE267661). To validate the CUT&Tag findings, we performed site-specific chromatin immunoprecipitation (ChIP)–qPCR on the NSUN2 promoter using primers spanning the H3K18la peak identified by CUT&Tag. Consistent with our genome-wide data, exogenous lactate treatment enriched H3K18la occupancy at the NSUN2 promoter (Fig. 6D), following previous protocols [35]. Importantly, the addition of lactate up-regulated the expression levels of H3K18la, NSUN2, and AKAP2 in ECs (Fig. 6E and F), whereas their down-regulation was dose-dependently achieved by glycolytic inhibitors (Fig. 6G). As an alternative loss-of-function strategy, we therefore silenced the principal H3K18 lactyltransferase EP300 with 2 independent siRNAs [36]. Depletion of EP300 decreased global H3K18la levels (Fig. 6H and Fig. S8A and B) and, more importantly, reduced NSUN2 mRNA and protein expression by ~60% compared with nontargeting control siRNA (Fig. 6H). Moreover, the exogenous supplementation of lactate could increase the expression of EP300, which agrees with previous observation [37]. These data indicate that EP300-mediated H3K18 lactylation is necessary for full activation of NSUN2 in ECs, thereby establishing a causal link between H3K18la and NSUN2 transcription. More interestingly, database analysis revealed a robust positive correlation between the lactate-producing enzyme lactate dehydrogenase A (LDHA) and NSUN2 (*R* = 0.803, *P* = 9.20 × 10^−8^; Fig. 6I), while no such association was observed for LDHB (*R* = 0.263, *P* = 0.160; Fig. S9A), suggesting that predominant lactate production by LDHA leads to transcriptional activation of NSUN2.

To further verify this hypothesis, we depleted the protein levels of LDHA and LDHB in 2 ECs (Fig. 6J), resulting in a significant decrease in the expression levels of H3K18la, NSUN2, and AKAP2 with LDHA knockdown. However, LDHB inhibition showed less inhibitory efficacy (Fig. 6K). Notably, the reintroduction of sodium lactate (NALA) exhibited a pronounced ability to counteract this downward trend (Fig. 6K).

Taken together, these results support that LDHA-mediated histone lactylation enhances the excessive expression of NSUN2.

**Fig. 7.**
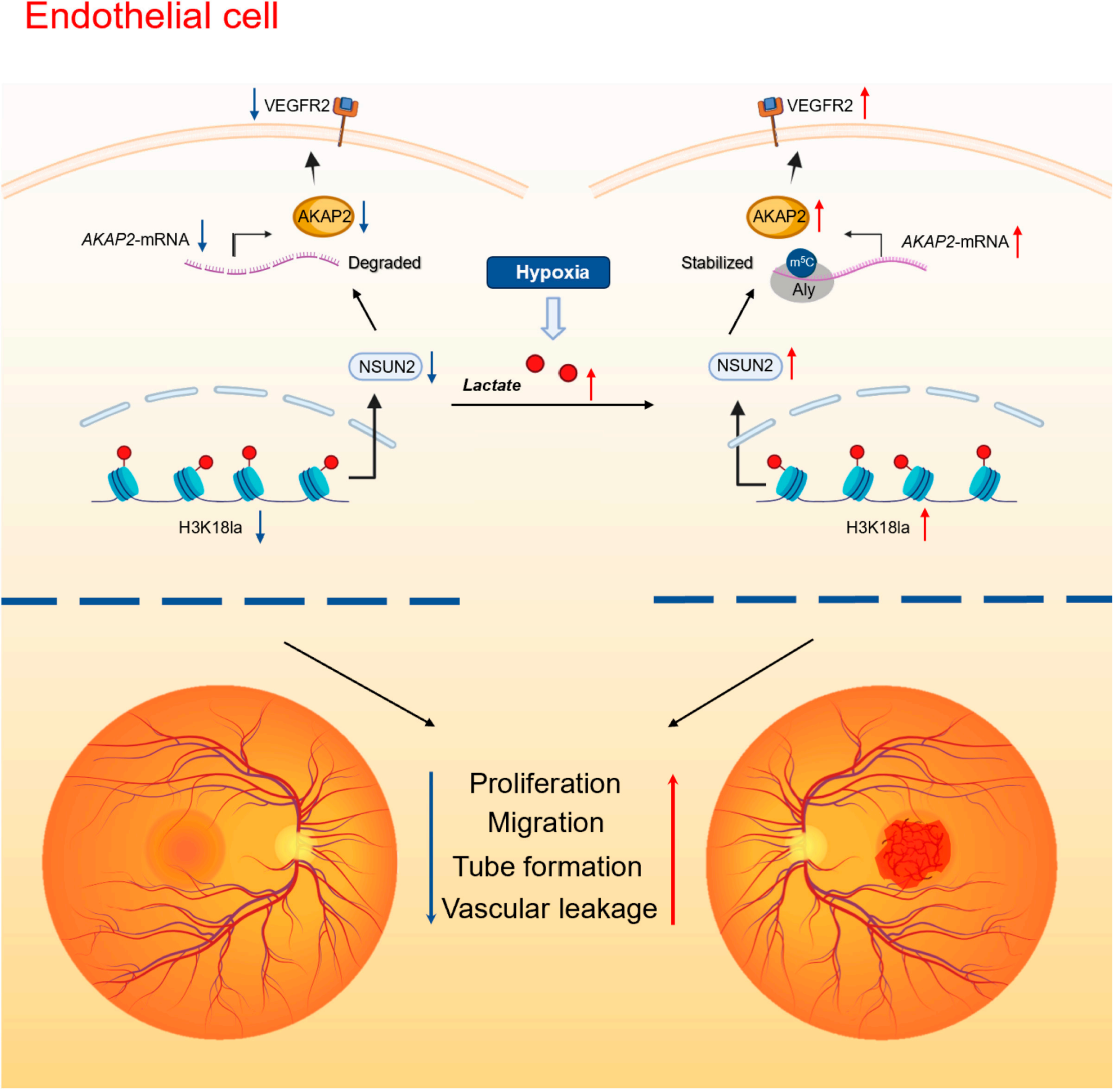
Schematic diagram of this study. Activation of transcription by H3K18la in ECs resulted in an increase in m^5^C methylation mediated by NSUN2, leading to enhanced expression of AKAP2 and VEGFR2 proteins, ultimately contributing to the progression of CNV.

## Discussion

In this study, we elucidate the pathological mechanisms of NSUN2-dependent m^5^C modification in CNV progression. Our results demonstrate that histone lactylation-induced NSUN2 up-regulation elevates m^5^C modification levels in CNV models. Compared to EC *Nsun2*^flox/flox^ controls, *Nsun2*-deficient mice (EC *Nsun2*^−/−^) exhibited reduced retinal vascular leakage following laser-induced CNV. Mechanistically, EP300-mediated H3K18 lactylation activates NSUN2, leading to increased m^5^C methylation on AKAP2 mRNA. This enhanced methylation stabilizes the AKAP2 mRNA through recognition by the m^5^C reader protein ALYREF, ultimately up-regulating VEGFR2 expression and promoting CNV development. These findings clarify a histone lactylation-NSUN2-AKAP2 axis as critical role for CNV pathogenesis and connect the signal cascade from metabolic microenvironment to posttranscriptional regulation, revealing promising therapeutic tactics for CNV-related disorders (Fig. 7).

NSUN2 is playing an increasingly pivotal role in endothelial dysfunction under pathological conditions. NSUN2 promotes vascular endothelial inflammation by methylating intercellular adhesion molecule 1 mRNA to mediate endothelial-leukocyte adhesion [38]. Beyond leukocyte, NSUN2 mediates T cell migration to endothelial in abdominal aortic aneurysm by increasing endothelial autotaxin expression [39]. In addition to accelerate inflammation, NSUN2 mediates endothelial pathological proliferation in an m^5^C-dependent manner in hypoxia condition, especially neoplastic neovascularization and hypoxic pulmonary hypertension (PH) pulmonary vascular remodeling [40–42]. By delineating the upstream histone lactylation modification and downstream up-regulation of VEGFR2 receptor expression, we have further defined the pathway through which NSUN2 contributes to endothelial dysfunction in pathological hypoxia.

Accumulating evidence demonstrates a close interplay between histone epigenetic modifications and RNA methylation [43–45]. Histone modifications could directly modulate the translation or recruitment of RNA methylation-associated enzymes by fine-tuning regional chromatin architecture, thereby establishing one facet of histone–RNA crosstalk [44,46]. Among these, histone lactylation, a histone modification involving the covalent attachment of a lactyl group to lysine residues on histone tails [24], is a key mechanism within this interaction. It has been verified to promote tumor malignant proliferation in an m^6^A- or m^1^A-dependent manner in malignant tumors characterized with abundant neovascularization [17,23,47]. Our study refines this histone–RNA regulatory axis in pathological angiogenesis. Meanwhile, complementing prior research on m^6^A and m^1^A modifications, this work expands current understanding of RNA methylation’s role in CNV biology. Critically, delineating crosstalk between distinct RNA methylation pathways, each exerting profound effects on transcriptional and translational regulation, remains essential to establish an integrated network-based mechanistic framework for this multifaceted regulatory paradigm.

AKAP2, a scaffolding protein that isolates PKA to specific subcellular locations by binding to its regulatory subunit, potentially functions to coordinate multiple components of the signal transduction pathway [48,49]. Evidence indicates that the AKAP2–PKA signaling complex promotes VEGFA expression and supports angiogenic protection in myocardial infarction [30]. In this study, we demonstrated that AKAP2 acts as a downstream pro-angiogenic effector of NSUN2 in CNV and further showed that AKAP2 regulates VEGFR2 expression in ECs. Based on these findings, we hypothesize that NSUN2-mediated stabilization of AKAP2 mRNA may partially explain the suboptimal responses or acquired resistance to anti-VEGF therapies observed in certain AMD patients, which deserves further investigation.

The H3K18la–NSUN2–AKAP2 axis presents novel therapeutic opportunities for CNV and other vascular proliferative disorders, potentially offering unique benefits for patients with suboptimal responses to current anti-VEGF therapies. Given the initiating role of lactate, strategies targeting LDHA warrant exploration for CNV treatment [50]. Meanwhile, NSUN2 is undoubtedly a promising therapeutic target for pathological angiogenesis, although no clinically approved NSUN2-specific inhibitors are currently available. Furthermore, this multi-level regulatory axis suggests that combination targeting strategies could yield synergistic effects in the future.

Nonetheless, several limitations of our study must be acknowledged, as they currently constrain its clinical translation. Although our study provides a comprehensive characterization of lactate-driven CNV pathogenesis via the NSUN2–AKAP2–VEGFR2 axis, identification of the precise m^5^C-modified sites on AKAP2 mRNA would enhance this work and its translation. However, given the absence of a unified consensus motif for m^5^C methylation, we have identified multiple candidate sites on AKAP2, each requiring functional validation through site-directed mutagenesis and RNA stability assays, which will be the focus of our future studies.

## Methods

### CNV model and EC-specific NSUN2-knockout animals

C57BL/6J mice (6 to 8 weeks old) were obtained from the Model Animal Research Center of Shanghai Jiao Tong University School of Medicine. All mice were housed in a specific pathogen-free environment. After deep anesthesia and dilation of the pupils, 3 to 5 laser photocoagulations (532 nm, 475 mW, 50 ms, and 50-μm spot size) on the right eye around the optic nerve of each mouse were performed to generate the CNV model. The presence of a bubble at the site of laser injury indicates the rupture of Bruch’s membrane and confirms the successful establishment of the CNV model. Animal ethics were approved by the Institutional Animal Care and Use Committee of the Ninth People’s Hospital, Shanghai Jiao Tong University School of Medicine (SH9H-2024-A951-SB). The conditional *Nsun2* gene-targeted mice (C57BL/6J) were obtained from Cyagen. The following primers were utilized for PCR amplification: F1: GTGCCTACATGATCTCTGAGGATG and R1: GTGGAGTTACAAGTGAACAGGAGG; F2: GAGTCACATCAAAGCCCTTGTTC and R2: AGCTATGCAGACTGAAGAATGAAG. The *Nsun2*^flox/flox^ mice were subsequently bred with Cdh5-Cre transgenic mice to generate *Nsun2*^flox/flox^ Cdh5-Cre mice for experimental purposes.

### Cell culture and transduction

HUVECs were purchased from ScienCell (USA). Primary human CEC cultures were obtained as previously described [51]. The use of human donor eyes and the approval for the use of human tissues were approved by the Ethics Committee of Shanghai JiaoTong University (approval number: SH9H-2019-T185-2). Briefly, the donor eyes from Ninth People’s Hospital, Shanghai JiaoTong University School of Medicine were prepared in a sterile environment, with the anterior segment and retinal neuroepithelial layer stripped. The pigment in the cortex is removed using 0.2% trypsin digestion, followed by separation of the choroid through 0.2% trypsin and 0.1% collagenase digestion. After filtration, ECs are purified using anti-CD31 magnetic beads and cultured under standard conditions.

ECs were cultured in EC medium (ScienCell, USA) containing 5% fetal bovine serum (ScienCell, USA), 1% EC growth supplement (ScienCell, USA), and 1% penicillin/streptomycin (ScienCell, USA) at 37 °C under 5% CO_2_ in a humidified atmosphere. Conditioned medium was collected from primary CECs after 24 h serum-free culture. For hypoxia treatment, ECs were maintained in complete medium at 5% O_2_ for 48 h before collection. Lactate (Topscience, China), 2-deoxy-D-glucose (2-DG) (Topscience, China), oxamate (Topscience, China), H-89 (Topscience, China), and 8-bromo-cAMP (Topscience, China) were used in cell culture of this study.

shRNAs targeting NSUN2, LDHA, LDHB, ALYREF, EP300, and AKAP2, along with an AKAP2 overexpression plasmid, were purchased from Genomeditech Corporation. The cells were transfected in accordance with the manufacturer’s instructions. The oligonucleotide sequences employed are presented in Table S1.

### RPE–choroid complex and fluorescence imaging

As previously described [52], the eye balls were carefully removed after the mice were euthanized. The remainder after lens separation was fixed in the formaldehyde–acetic acid solution (FAS) eyeball fixative solution (Servicebio, China) at room temperature for a duration of 3 h. The RPE–choroid complex was gently detached for total protein and RNA extraction.

For fluorescence imaging, the RPE–choroid complex was blocked with 5% goat serum in phosphate-buffered saline–Tween 20 (PBST) for 1 h, followed by staining overnight at 4 °C using DyLight 594 Labeled GSL l-isolectin B4 (Maokang Bio, China) or CD31 (Proteintech, China). After cleaning the complexes with a blocking solution, images were acquired and quantified using a fluorescence microscope (Nikon, Japan).

### Fundus examinations

As previously described [53], following anesthesia and pupil dilation, mice were intraperitoneally administered 2.5 ml/kg of a 10% sodium fluorescein solution (Alcon, China). Sequential fluorescein angiography (FA) and IR photographs of the posterior eye segment were captured at 1 min post-injection using the Heidelberg Spectralis HRA system (Heidelberg Engineering Inc., Germany). The optical coherence tomography (OCT) system (Heidelberg, Germany) was used to monitor the changes in retinal thickness.

### Statistical analysis

Quantification data are presented as the mean ± SD. Prism 9.0 software (GraphPad, USA) was used for statistical analyses, and differences between 2 groups were compared with an unpaired Student’s *t* test. Differences were considered significant at *P* < 0.05, and asterisks denote statistical significance.

## Data Availability

Original sequencing data of RNA-seq and meRIP-seq are accessible in the GEO (GSE267662). The raw data of histone lactylation CUT&Tag have been deposited in GEO under the accession number of GSE267661. The correlation analysis was created in R2 Genomics Analysis and Visualization Platform (https://hgserver1.amc.nl/), by using genome-wide mRNA expression profiling data in the 30 ocular ECs (GEO, GSE144785).
