# Supplementary material for: Lactylation-Boosted m5C RNA Modification Drives Choroidal Neovascularization
Source: Research (Wash D C). 2025 Oct 2;8:0913. doi: 10.34133/research.0913 (PMC12489183; doi:10.34133/research.0913)
Supplement: Supplementary 1 — Materials and Methods Figs. S1 to S10 Tables S1 to S4 [file research.0913.f1.docx]

Supplementary Materials for

**Lactylation-boosted m5C RNA Modification Drives Choroidal Neovascularization**

Sipeng Zuo *et al.*

^*^Corresponding author. Email: Xianqun Fan; fanxq@sjtu.edu.cn, Peiwei Chai; chaipeiwei123@sjtu.edu.cn and Linna Lu; linatutu@sjtu.edu.cn

**This file includes:**

Supplementary materials and methods

Figs. S1 to S10

Tables S1 to S4

**Supplementary materials and methods**

**Single-cell RNA sequencing (scRNA-seq) data analysis**

scRNA-sequencing raw data deposited in the ArrayExpress under accession number E-MTAB-8119 was analyzed. Cell-type identification and cluster analysis were performed through the Seurat program (version 4.1.0), with the cluster analysis parameter resolution set to 0.6. Then, the Seurat program was used to identify differentially expressed genes (DEGs) between different samples or clusters. 4.1.0 (version) with the standard of | log2 (fold change) | > 0.5, P < 0.05).

**RNA extraction, mRNA isolation and qRT-PCR**

This procedure was performed as described previously[1]. Briefly, total RNA was extracted using TRIzol reagent (Takara, Japan). The mRNA was subsequently extracted in accordance with the guidelines. The cDNA was synthesized using a PrimeScript RT Reagent Kit (Takara, Japan), followed by qRT‒PCR analysis performed on a Roche LightCycler 480 system (ABI, USA). The primer sequences utilized in this study can be found in Table S2.

**Dot blot assay**

As previously described[1], mRNA was quantified and diluted in 10 mM Tris-EDTA buffer. The designated amounts of RNA samples were loaded onto Hybond-N + membranes (Beyotime, China). After a brief drying process, the membrane was crosslinked at 254 nm UV for 1 min. Following this step, it was blocked with 5% milk for 1.5 hours at room temperature and incubated overnight at 4 °C with an anti-m^5^C antibody (Abcam, USA). After three washes with TBST (Thermo Fisher Scientific, USA), the membranes were incubated with HRP-conjugated anti-rabbit IgG (Proteintech, China) for 1.5 hours at room temperature and then visualized using an ECL kit (Thermo Fisher Scientific, USA) along with a detection instrument (Tanon Science, China).

**Western blotting analysis**

Total protein was extracted from treated cells and subsequently sonicated in RIPA lysis buffer (Thermo Scientific, USA), which contained a mixture of 1% protease and phosphatase inhibitors (NCM biotech, China). The protein concentration was determined using the BCA protein assay kit (Thermo Scientific, USA). The equal amounts of obtained supernatant were separated on SDS-PAGE gels (Bio-Rad, USA) and transferred onto PVDF membranes (Millipore, USA). Blocking was performed in 5% milk for 1.5 hour at room temperature. Subsequently, incubation with the following primary antibodies was carried out overnight at 4°C: NSUN2 (Proteintech, China), β-actin (Proteintech, China), AKAP2 (Abcam, China), ALYREF (Abcam, USA), Histone H3 (Proteintech, China), Pan-Kla (PTM Bio, China), H3K18la (PTM Bio, China), LDHA (Proteintech, China), LDHB (Proteintech, China) and VEGFR2 (Proteintech, China). Finally, detection was performed using HRP-conjugated secondary antibodies (Proteintech, China) followed by signal detection performed using an ECL kit (Thermo Fisher Scientific, USA) and visualized with the imaging system (Tanon, China).

**Cell proliferation assay**

Approximately 1-3 × 10^3^ndothelial cells were seeded in 96-well plates, and at the indicated time points, 10 μL of Cell Counting Kit-8 reagent (Dojindo, Japan) was added to each well. Following a 2-hour incubation period, the absorbance at 450 nm was measured using a microplate reader (BioTek, USA).

**Tube formation assay**

The matrigel (Corning, USA) was incubated for 30 minutes at 37 °C until solidified. Subsequently, the appropriate number of endothelial cells were seeded on matrigel. Random images were captured 4–6 hours after CD31 immunofluorescence staining was performed on the cells, and the quantification of tube formation was performed using image J software.

**Transwell migration assay**

Endothelial cells were seeded into transwell chambers with a pore size of 8-μm, and the chambers were subsequently placed in 24-well plates for culture. After 24 hours, the cells were stained with crystal violet solution (0.1%, Beyotime, China) for a duration of 1 hour. Random images were captured, and cell counts were conducted using image J software.

**Chorioallantoic membrane assay**

The CAM assay was performed utilizing the 8-day old fertilized chicken embryos as previously described[2]. A small hole was created on the surface of the air sac to expose the CAM. A volume of 150 μL of conditioned medium was carefully dropped onto the membrane and the hole was subsequently covered with sterile tape. After 72 hours of incubation, images of CAMs were captued to quantify the angiogenic response.

**RNA-Seq and MeRIP-Seq**

The integrity of total RNA was assessed using a 2100 Bioanalyzer from Agilent Technologies, while the concentration was determined using a Qubit 2.0 fluorometer with the Qubit RNA assay kit from Invitrogen. Subsequently, sequencing libraries were prepared utilizing the Illumina TruSeq RNA Sample Prep Kit (Illumina, USA), and high-throughput sequencing was performed on an Illumina HiSeq 3000 platform (Illumina, USA).

The m^5^C-MeRIP-seq experiment was conducted by Cloudseq Biotech (Shanghai, China). Briefly, RNA was randomly fragmented and incubated overnight with protein A/G beads conjugated to m^5^C antibodies. Subsequently, the captured RNA was eluted and extracted using TRIzol reagent. Library construction was performed for both input samples and m^5^C IP samples. The libraries were assessed using an Agilent 2100 Bioanalyzer (Agilent, USA) and sequenced on a NovaSeq 6000 platform (Illumina, USA). Following quality control and removal of low-quality reads, the clean reads from all libraries were aligned to the reference genome (HG19) using HISAT2 software (v2.0.4).

**Isobaric tags for relative and absolute quantitation (iTRAQ) proteomic analysis**

As previously described[1], the endothelial cells were lysed with SDT buffer (4% SDS, 100 mM Tris-HCl, 1 mM DTT, pH 7.6). An analysis of the protein supernatants and its identification and quantification using tandem mass tag (TMT) labeling, fractionation, and LC-MS was conducted.

**Cleavage under targets and tagmentation (CUT&Tag) and data analysis**

As previously described[3], the endothelial cells were incubated with concanavalin A-coated magnetic beads (Bangs Laboratories, USA) at RT for 10 min. The cells were then incubated with the secondary antibody (Millipore, USA) for 60 minutes, followed by incubation with the pA-Tn5 adaptor complex for an additional 60 minutes at room temperature. Subsequently, they were treated with tagmentation buffer (10mM MgCl2 in Dig-med buffer) for another 60 minutes at 37°C. DNA purification was performed using phenol–chloroform–isoamyl alcohol extraction and ethanol precipitation. After library amplification, sequencing was carried out using an Illumina NovaSeq 6000 system.

**Figs S1 to S10**


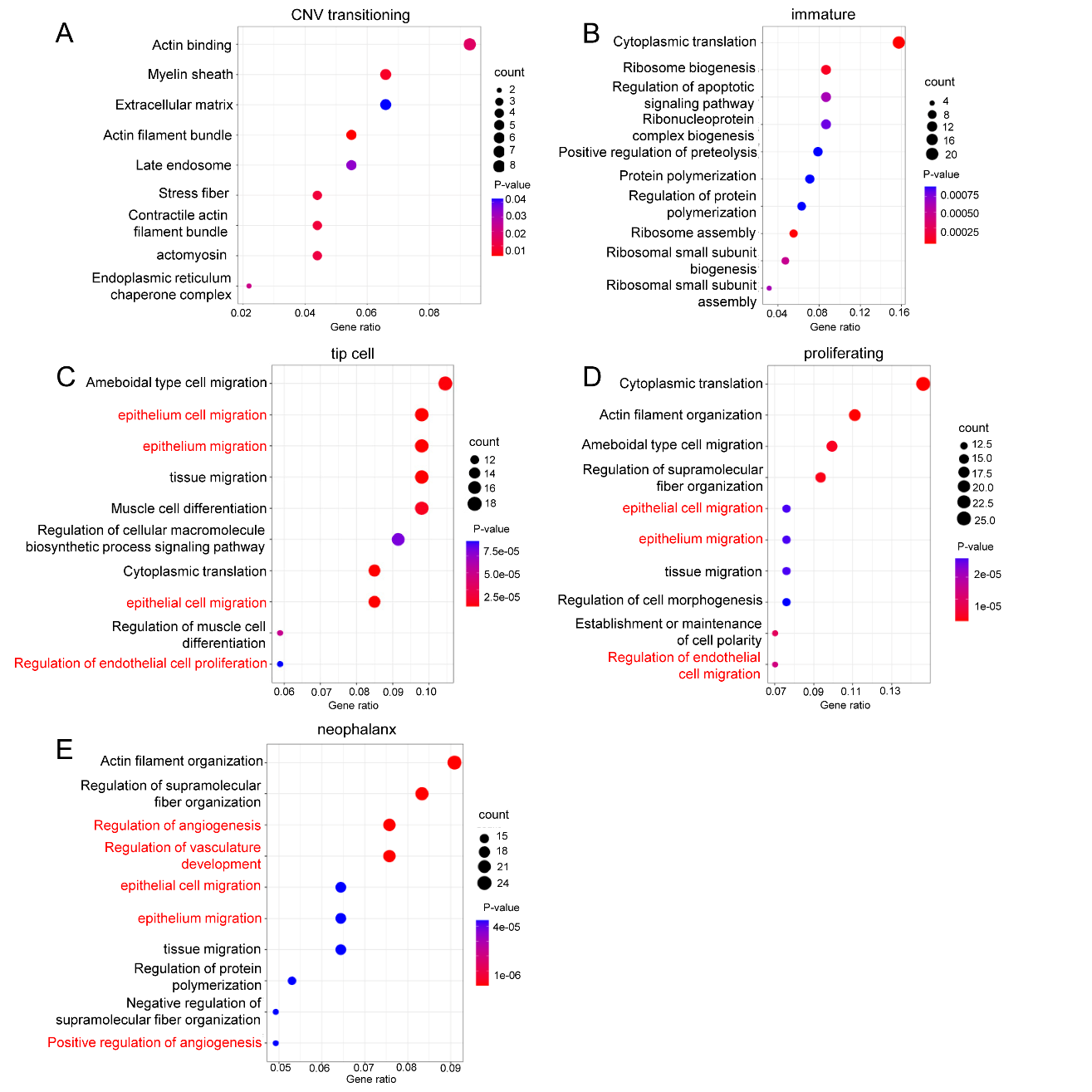


**Fig. S1.** **The numerous pathways were found to be upregulated in CNV.**

**(A-E)** GO analysis of differently expressed genes in various CNV-ECs clusters compared to healthy ECs.

**
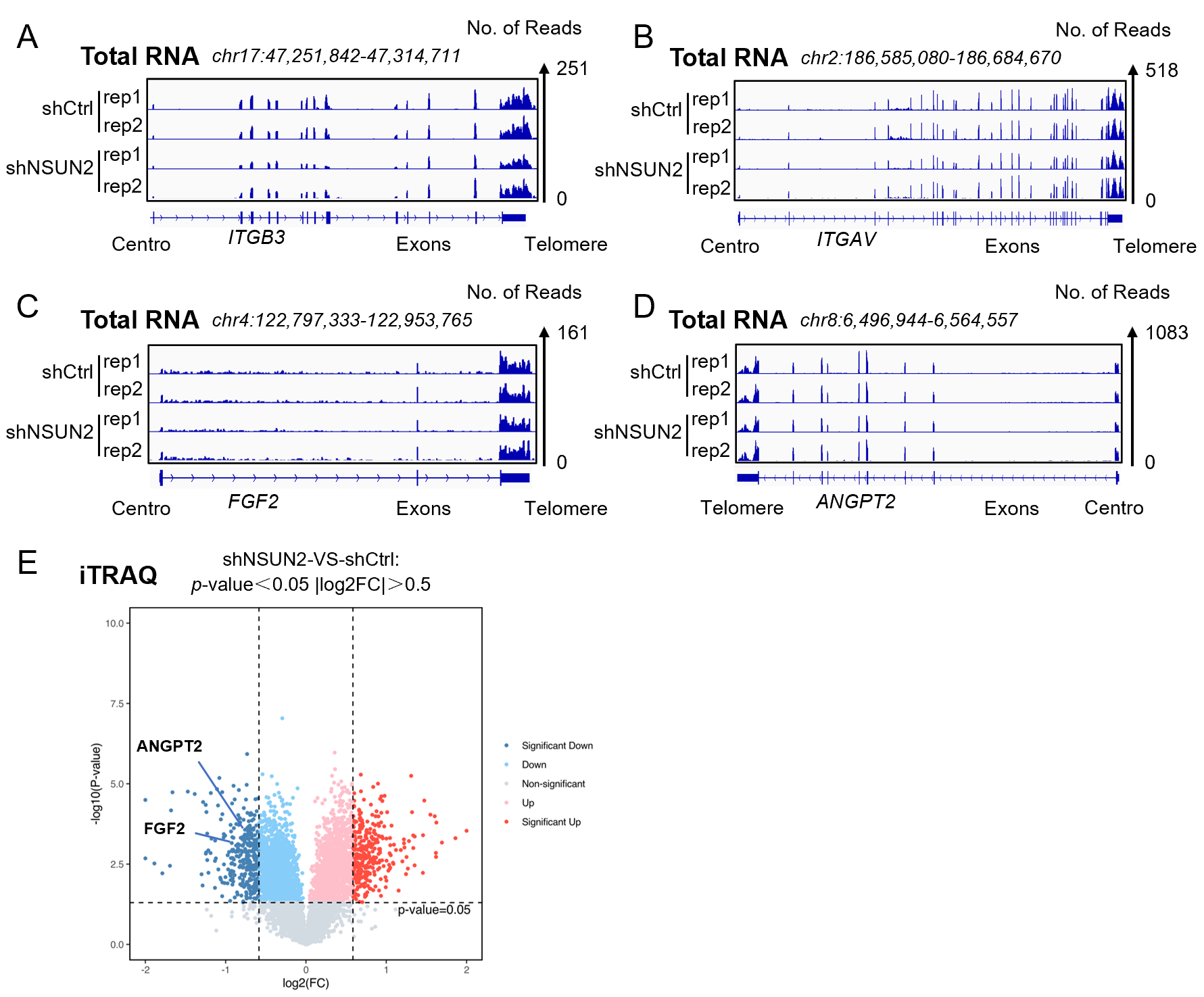
**

**Fig. S2.** **The pro-angiogenesis factors exhibited a lower expression level in the NSUN2-deficient ECs.**

**(A-D)** The RNA-seq result showing that the RNA expression levels of pro-angiogenesis factors (ITGB3, ITGAV, FGF2, ANGPT2) were lower in NSUN2-deficient ECs.


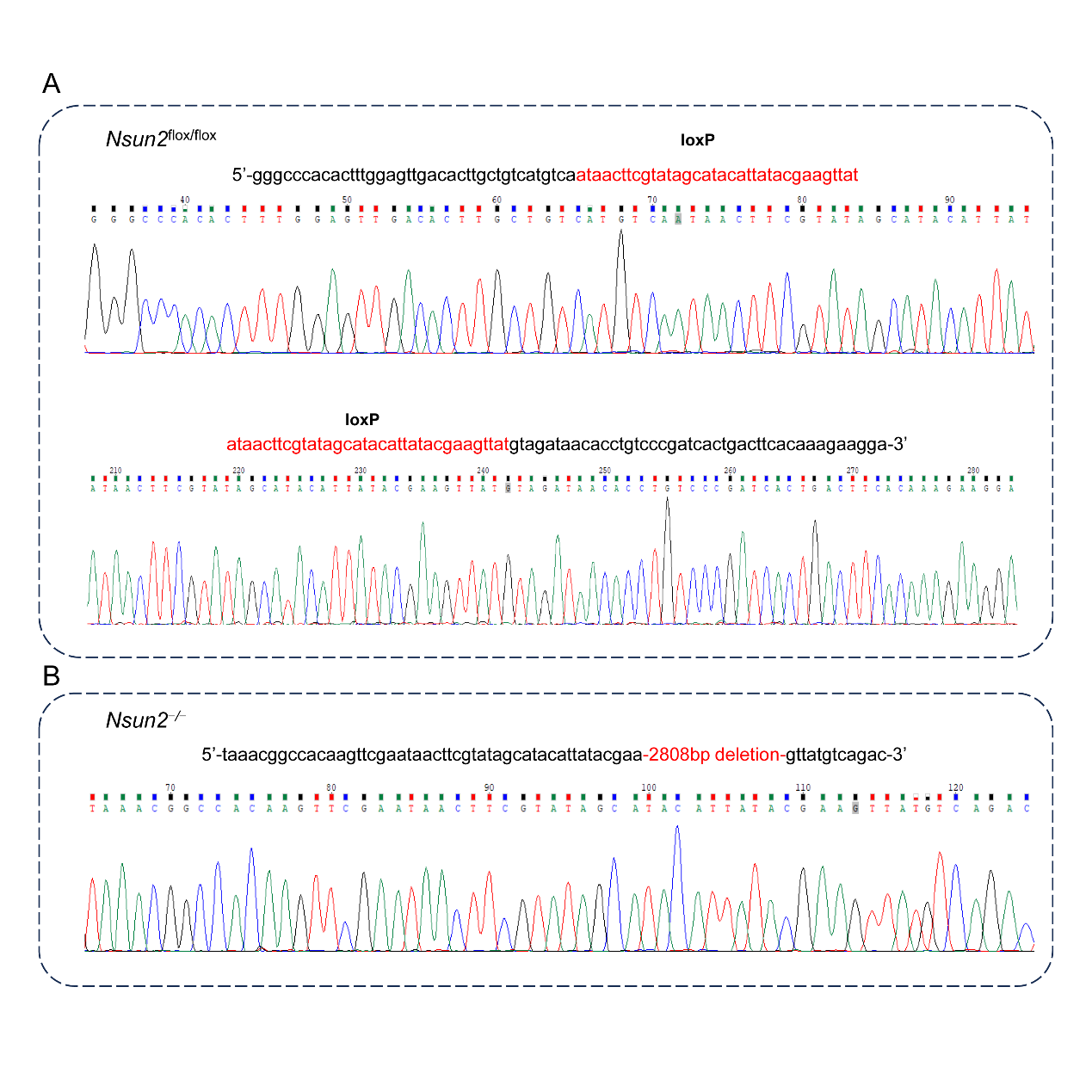


**Fig. S3.** **The conditional *Nsun2* gene-targeted mice were identified.**

**(A)** Sanger sequencing showing the DNA sequence of *Nsun2*^flox/flox^ mice.

**(B)** Sanger sequencing showing the DNA sequence of *Nsun2*^flox/flox^ Cdh5-Cre mice.


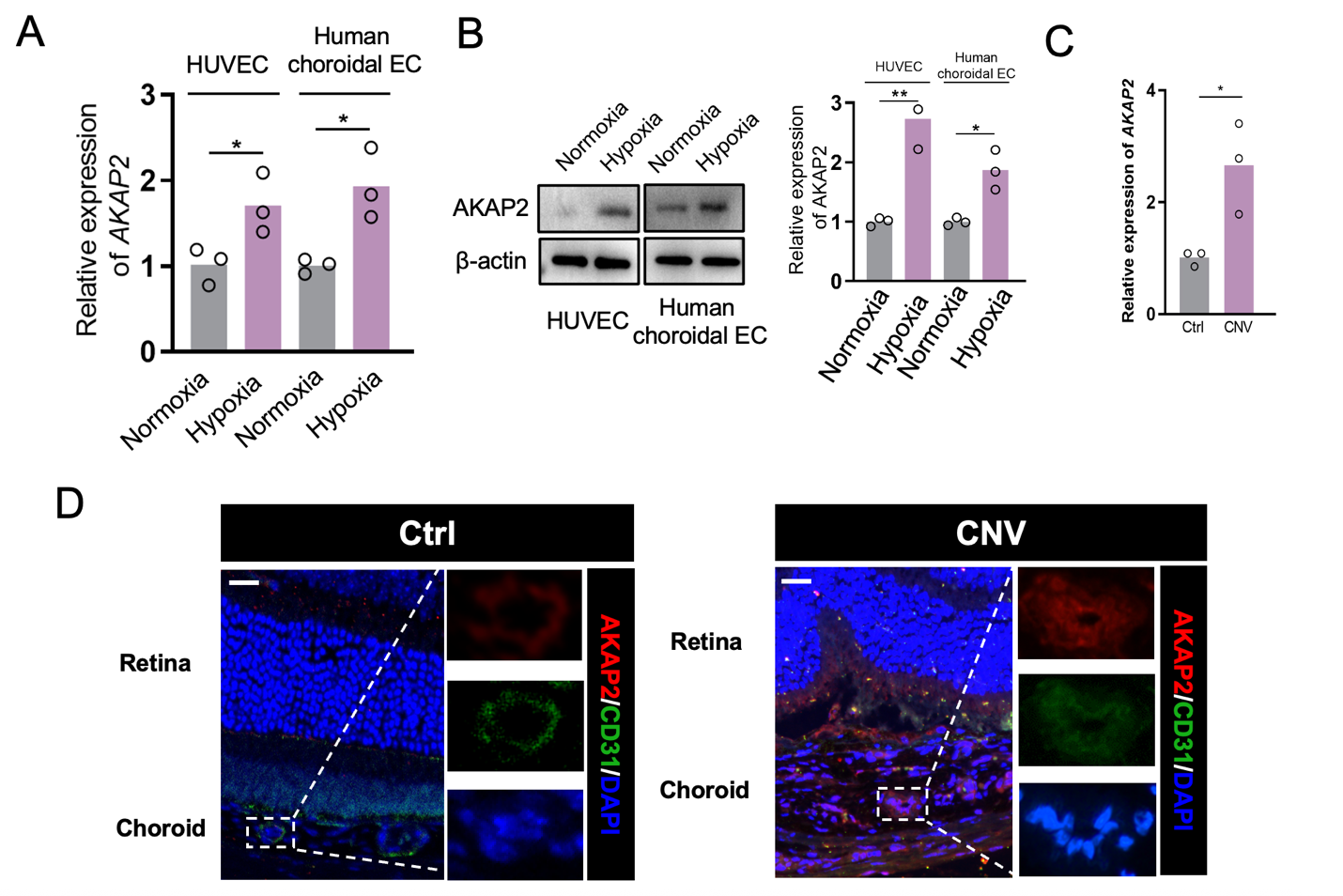


**Fig. S4.** **AKAP2 was upregulated in** **hypoxic ECs and CNV-bearing mice.**

**(A, B)** qPCR and WB assays showing AKAP2 expression in hypoxic ECs (n=3). The data are presented as the mean ± SD of experimental triplicates. Significance was determined by an unpaired two-tailed Student’s t test. *P < 0.05.

**(C)** qPCR showing AKAP2 expression in RPE-choroid complex of control and CNV-bearing mice (n=3). The data are presented as the mean ± SD of experimental triplicates. Significance was determined by an unpaired two-tailed Student’s t test. *P < 0.05.

**(D)** Immunofluorescence of AKAP2 (red), CD31 (green) and DAPI staining (blue) in control and CNV-bearing mice (n=3). Scale bars: 20 μm.

*
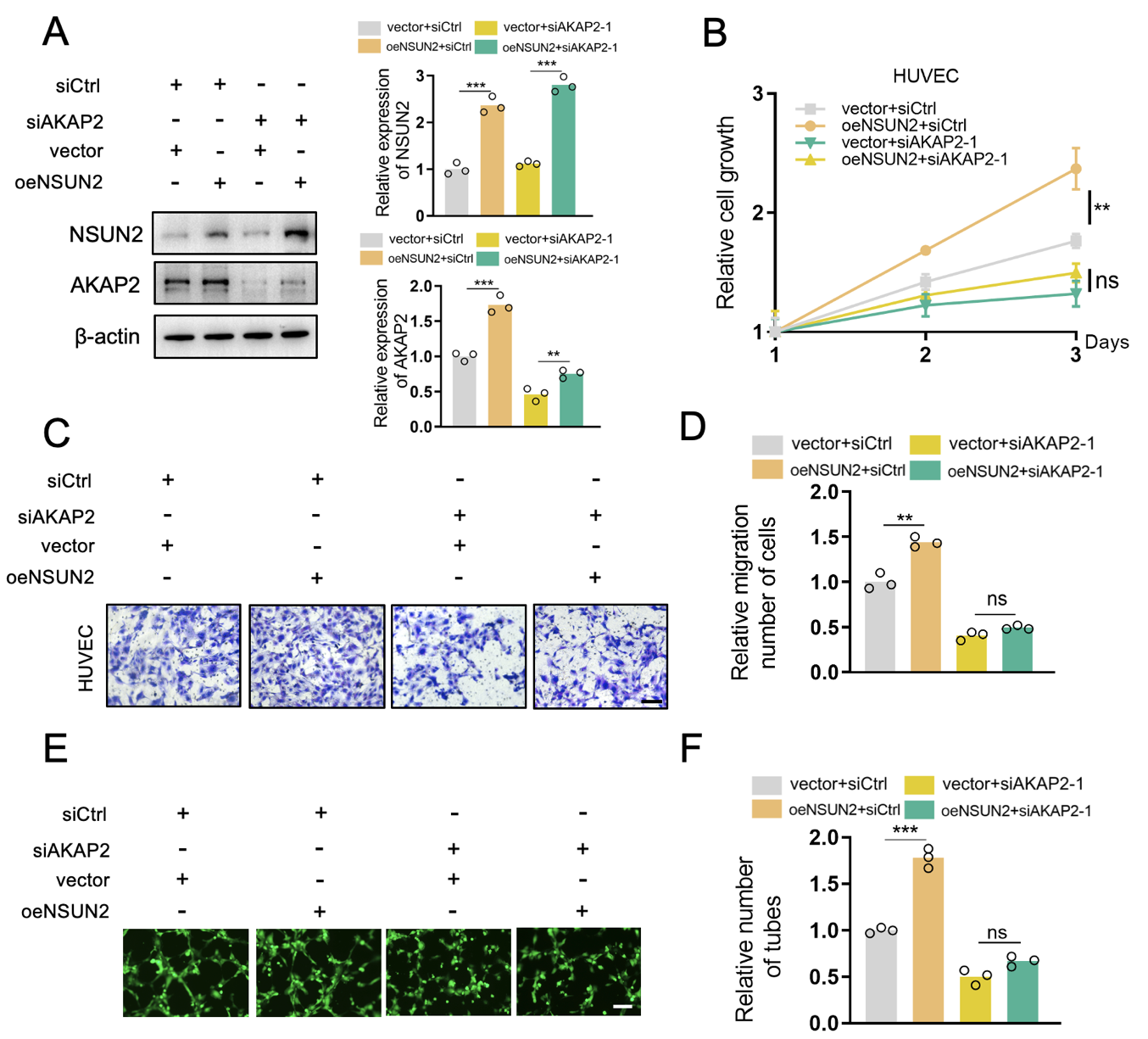
*

**Fig. S5.** **The pro-angiogenesis of NSUN2 depends on AKAP2 activation.**

**(A)** Western blot data showing NSUN2 expression in ECs following AKAP2 knockdown and NSUN2 overexpression.

**(B)** A CCK8 assay was performed to assess the proliferation of AKAP2-deficient ECs after NSUN2 overexpression. The data are presented as the mean ± SD of experimental triplicates. Significance was determined by an unpaired two-tailed Student’s t test. *P < 0.05, **P < 0.01.

**(C, D)** A transwell assay was employed to evaluate the migration of AKAP2-deficient ECs after NSUN2 overexpression. Scale bars: 100 μm. Representative images from three experimental replicates are shown. The data are presented as the mean ± SD. Significance was determined by an unpaired two-tailed Student’s t test. *P < 0.05, **P < 0.01.

**(E, F)** A tube formation assay was employed to evaluate the tube formation of AKAP2-deficient ECs after NSUN2 overexpression. Scale bars: 100 μm. Representative images from three experimental replicates are shown. The data are presented as the mean ± SD. Significance was determined by an unpaired two-tailed Student’s t test. *P < 0.05, **P < 0.01, ***P < 0.001.


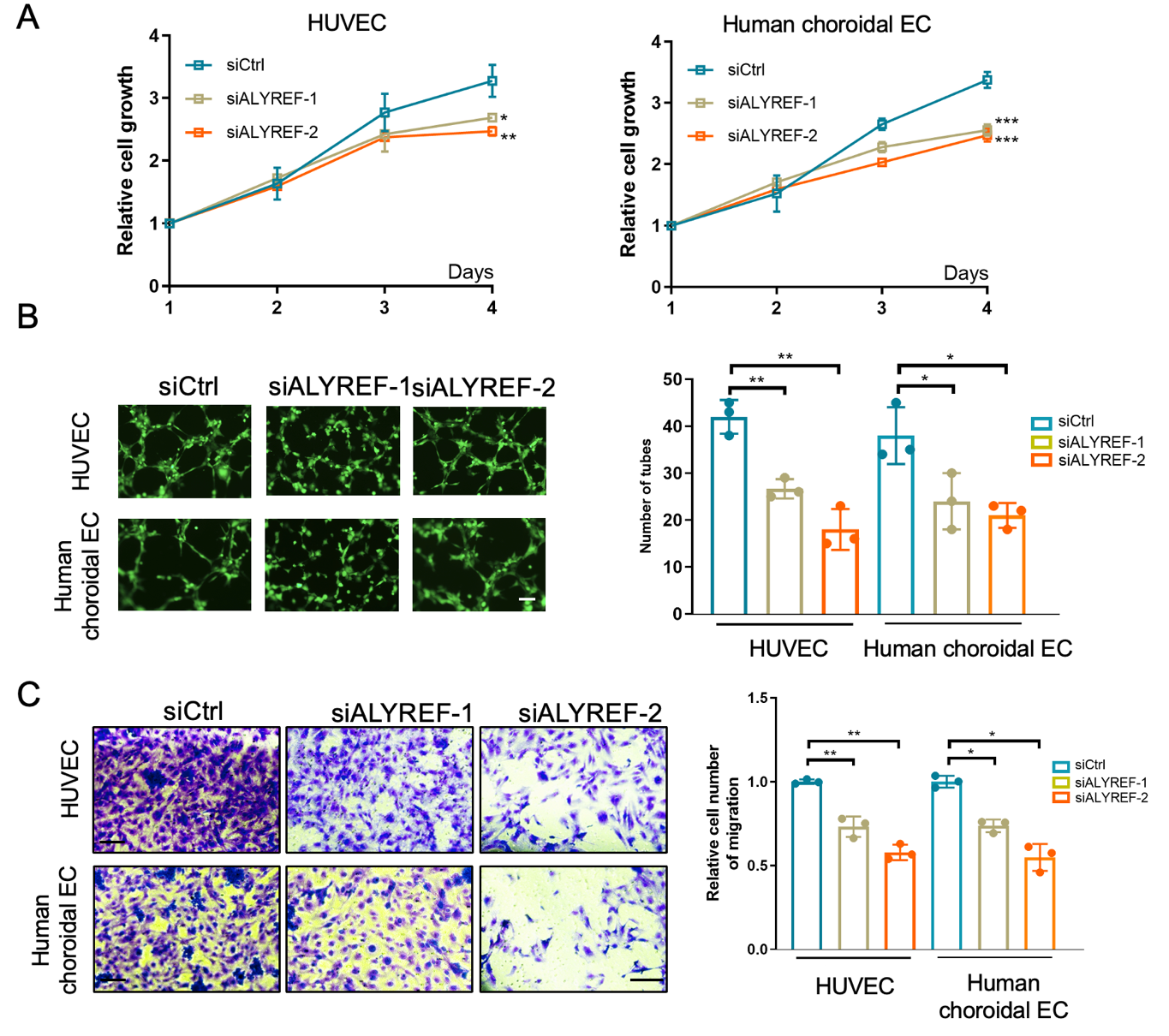


**Fig. S6.** **ALYREF knockdown impaired the proliferation, migration and tube formation ability of ECs.**

**(A)** A CCK-8 assay was employed to evaluate the proliferation of ECs after NSUN2 knockdown (n=3). The data are presented as the mean ± SD of experimental triplicates. Significance was determined by an unpaired two-tailed Student’s t test. *P < 0.05, **P < 0.01.

**(B)** A tube formation assay was employed to evaluate the tube formation of ECs following ALYREF knockdown (n=3). Scale bars: 100 μm. Representative images from three experimental replicates are shown. The data are presented as the mean ± SD. Significance was determined by an unpaired two-tailed Student’s t test. *P < 0.05, **P < 0.01.

**(C)** A transwell assay was employed to evaluate the migration of ECs following ALYREF knockdown (n=3). Scale bars: 100 μm. Representative images from three experimental replicates are shown. The data are presented as the mean ± SD. Significance was determined by an unpaired two-tailed Student’s t test. *P < 0.05, **P < 0.01.


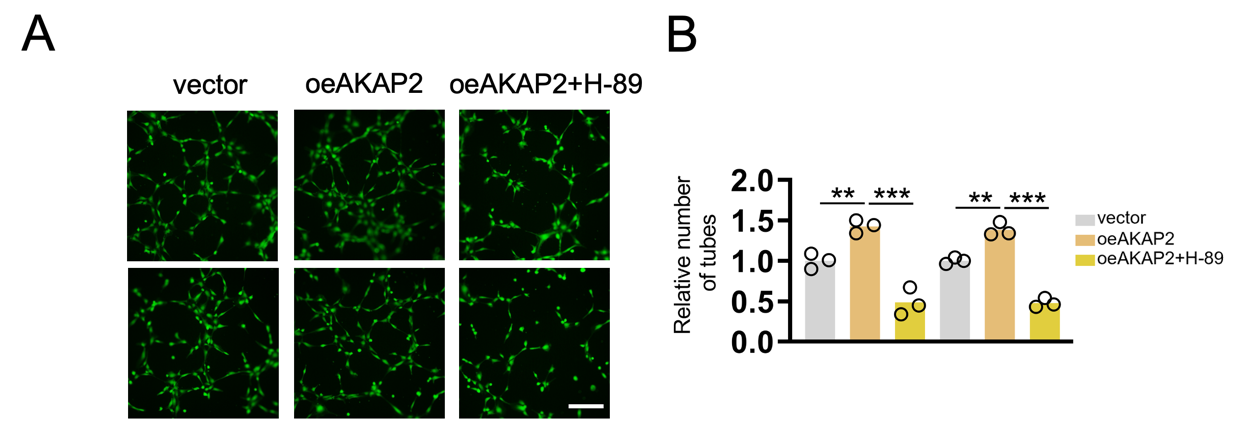


**Fig. S7.** **Tube formation assay and statistical analyse of AKAP2-overexpression and H-89 treatment.**

**(A, B)** A tube formation assay was employed to evaluate the tube formation of AKAP2-overexpressed ECs with H-89 treatment. Scale bars: 100 μm. Representative images from three experimental replicates are shown. The data are presented as the mean ± SD. Significance was determined by an unpaired two-tailed Student’s t test. **P < 0.01, ***P < 0.001.


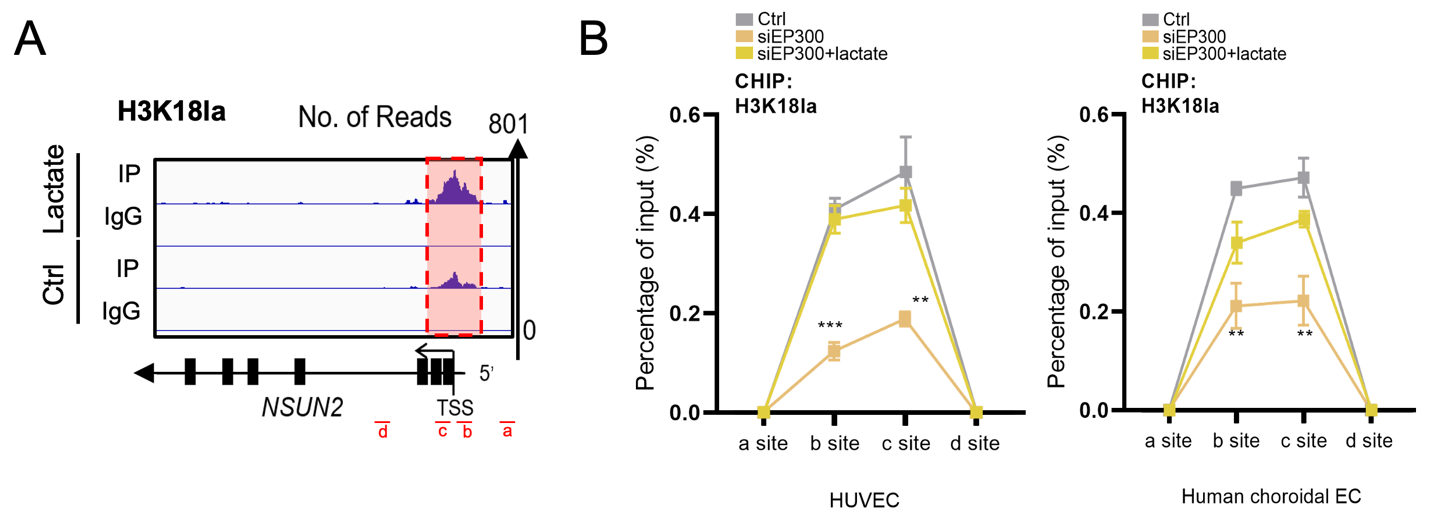


**Fig. S8.** **H3K18la alternations after EP300 knockdown.**

**(A)** IGV tracks from CUT&Tag assay showing H3K18la enrichment of NSUN2. Sites a–d are distributed in the NSUN2 genomic region, and sites b and c are the H3K18la peaks.

**(B)** CHIP-qPCR assay of H3K18la status in NSUN2 upon EP300 knockdown with lactate treatment (n=3). The data are presented as the mean ± SD of experimental triplicates. Significance was determined by an unpaired two-tailed Student’s t test. **P < 0.01, ***P < 0.001.


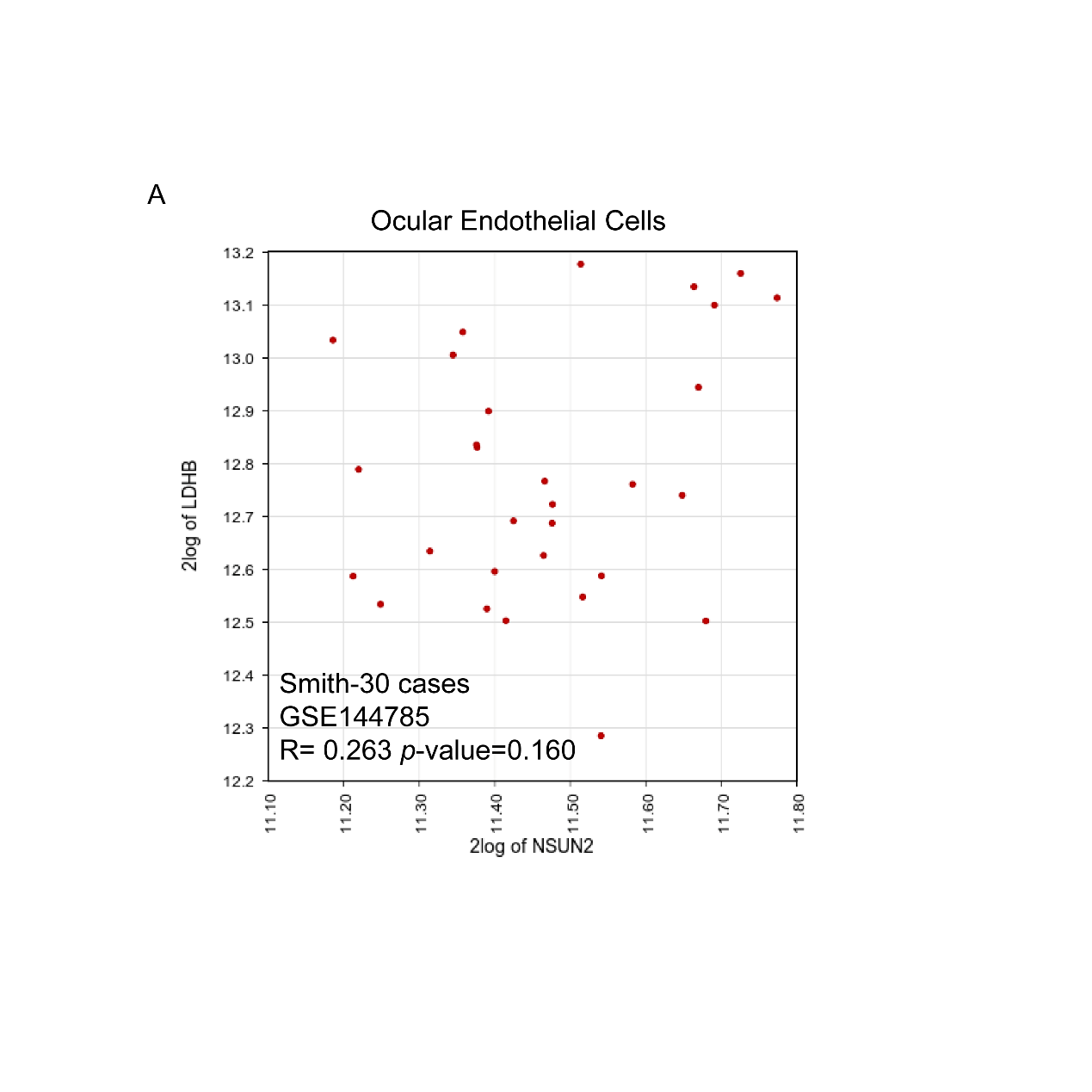


**Fig. S9.** **No significant relationship was presented between LDHB and NSUN2.**

**(A)** Correlation analysis between the lactate-producing enzyme LDHB and NSUN2 through GEO data.


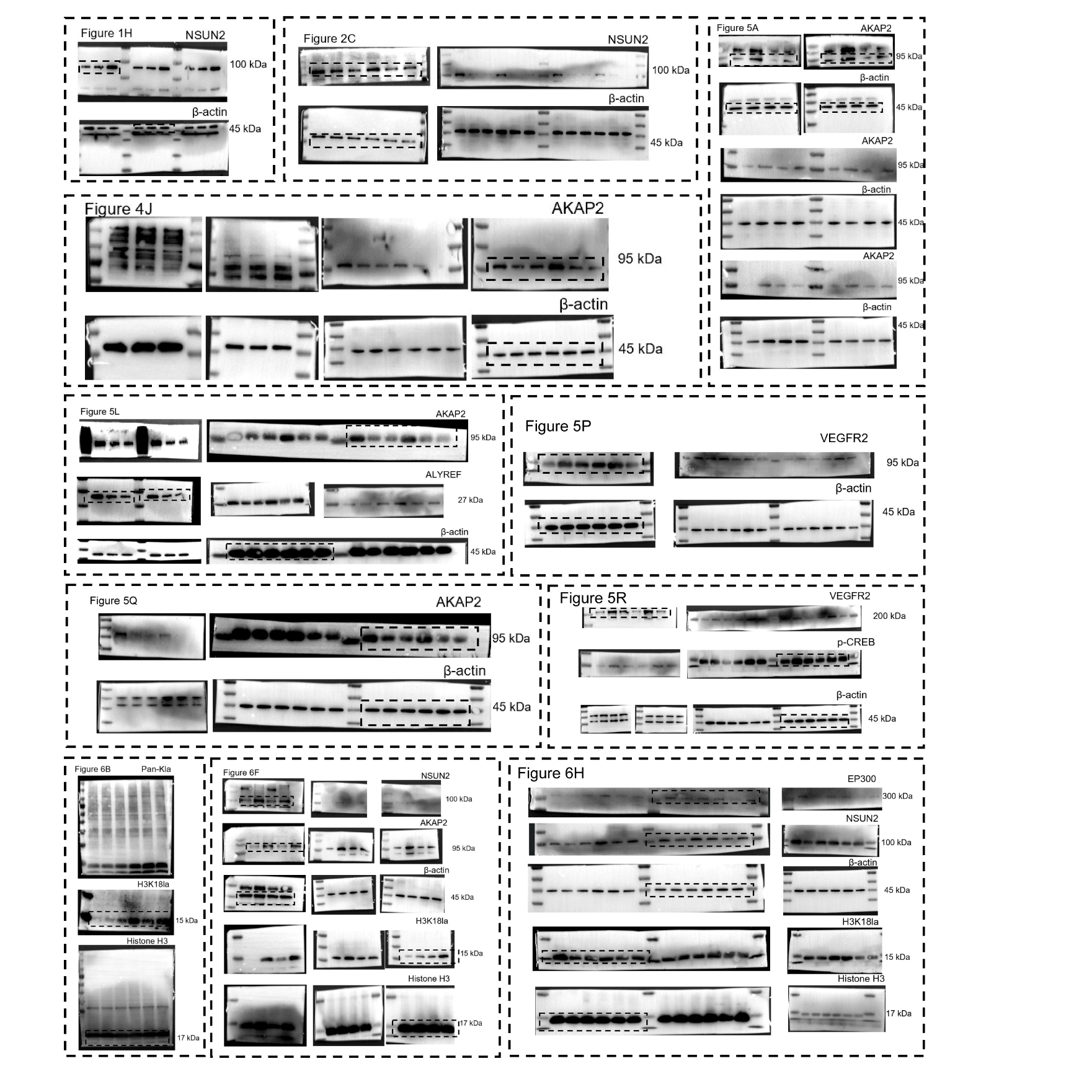


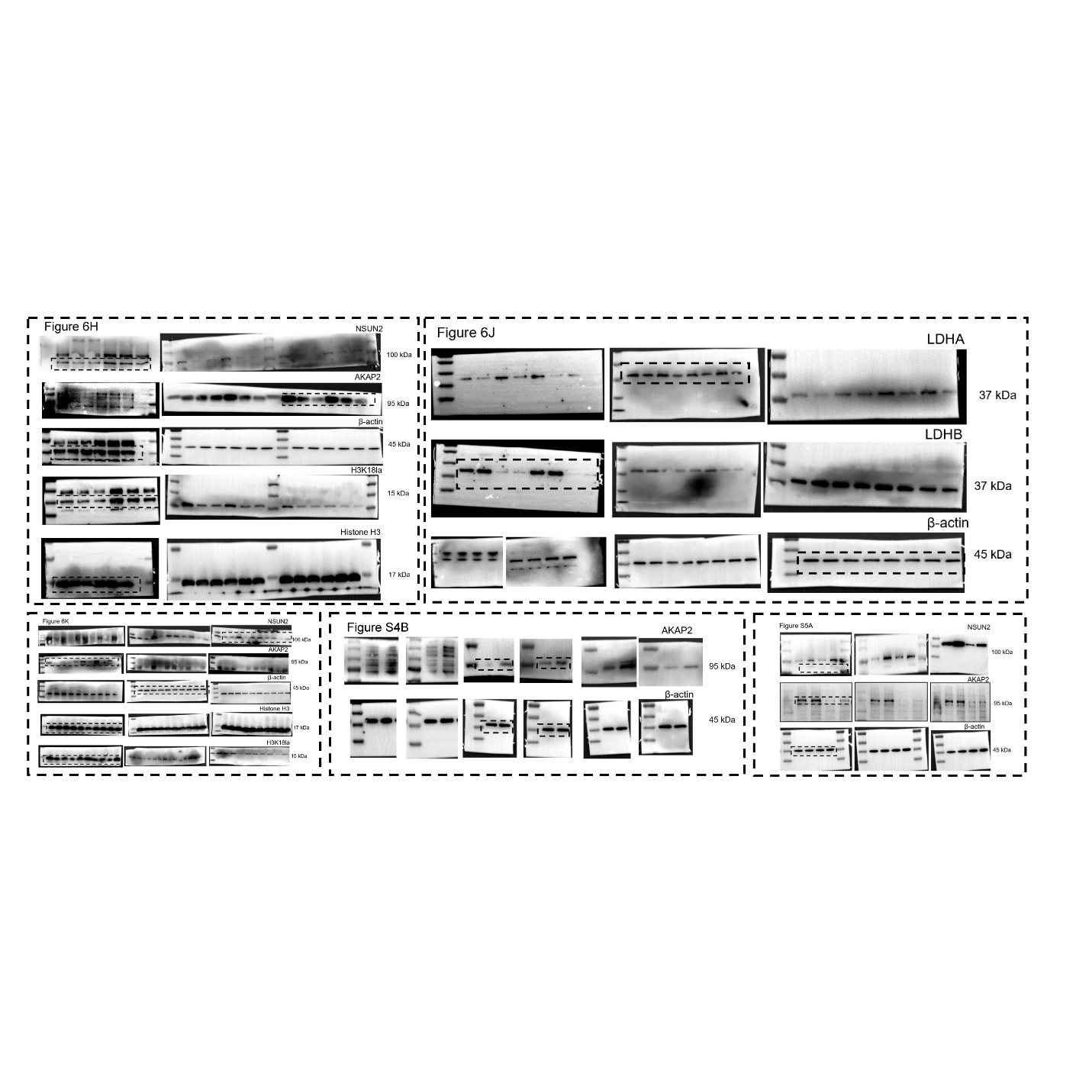


**Fig. S10.** **Unprocessed WB images.**

**Tables S1 to S4**

**Table S1. shRNA and siRNA used in the study**

| shRNA | Sequence (5′-3′) |
| --- | --- |
| shNSUN2-1  shNSUN2-2  shLDHA  shLDHB | CGAATGATGTGGACAACAA  TGAGAAGATGAAGGTTATTAA  CCACCATGATTAAGGGTCTTT  CGTGATTGGAAGTGGATGTAA |
| siRNA | Sequence (5′-3′) |
| siALYREF-1  siALYREF-2  siAKAP2-1  siAKAP2-2  siEP300-1  siEP300-2 | GGAAACTGCTGGTGTCCAATC  CGTGGAGACAGGTGGGAAACT  GCATCCAATGAGACAACCAAT  CAGCGGACTTTGTCCATGATT  CAATTCCGAGACATCTTGAGA  GCCTTCACAATTCCGAGACAT |

**Table S2. Primers used in the study**

| **Gene** | **Forwards (5′-3′)** | **Reverse (5′-3′)** |
| --- | --- | --- |
| NSUN2  AKAP2  ALYREF  β-actin | GCAAGCTCCAAAGCACCTTC  GCTGGACGAGGAACATCTGGAG  CCATGGCCGACAAAATGGAC  GAGCACAGAGCCTCGCCTTT | ATGTGGACGGCAGGAAAGAG  GCTGCTGCTGTAACTGCTTCT  CGGGAAGTTGTTTTGGCCTG  TCATCATCCATGGTGAGCTGG |

**Table S3. Proteins altered in HUVECs upon NSUN2 knockdown**

| **Accession** | **Gene** | **MW [kDa]** | **log_2_FoldChange** | ***P*-value** |
| --- | --- | --- | --- | --- |
| P19075  Q2M2I5  Q9NQW1  Q92622  Q6TDU7  P56159  P08729  Q8IWU6  Q9UPI3  Q8TDG2  Q9UKT9  Q8N841  Q8N370  P57055  A1L188  P02751  P0DOY2  Q9UBL6  Q0VDI3  Q01629  A6H8Y1  Q5YKI7  P61812  P01876  Q9NR99  Q8NCY6  Q9NQC1  Q8NHM4  Q02383  Q7Z3D4  P05787  P39060  Q9NR96  Q7Z7G0  P28906  P31151  P25445  P07585  P24394  Q5D862  P02462  Q15465  P41217  Q92633  Q8NEG7  Q14766  Q96HY7  P0DPH8  P61073  Q8IVL0  Q9NY26  Q5VUE5  Q58FG1  P27658  Q9BPX5  Q9HCY8  Q71UI9  Q9BVG8  O15232  Q8NFP9  Q52LD8  Q8TD31  P12110  P17661  P02787  P61626  Q9Y6V0  Q08J23  Q9UBX7  Q629K1  Q2KJY2  Q9UHI8  Q8IZE3  P08174  Q16270  Q3ZCU0  P30837  Q13296  Q9ULC0  Q9UPZ6  Q6ZUX7  P48668  Q04941  P34741  P62995  O14495  P54851  Q9Y4K0  Q3KR37  P01834  Q9H1C4  Q6UWH4  Q9UPY5  P05106  O14494  O00622  P21453  P20908  P02647  O95025  Q96DR8  O75528  P14543  P02749  O60687  O14763  P98160  Q02223  Q92485  Q0VAQ4  P02760  Q8WTV0  Q9BSV6  P30408  P78382  P38570  O95847  Q5T280  P05997  P08572  P23490  O00220  Q8IY34  Q9Y6R1  Q9NU53  P42167  P07477  Q8IUE6  Q96T49  Q8N1N4  Q9NVH6  P20930  Q9P291  P14210  Q9UM19  Q92985  P33947  B9A064  Q9Y618  O75324  P01023  Q9H920  Q8TDB4  P52926  Q92737  P21953  O60637  P42773  O75907  Q14112  P30511  Q8IVB5  P13498  Q7Z794  Q86VR2  Q96BD0  P49746  Q07617  Q9Y312  Q13686  Q8IVH2  Q8TDH9  Q2T9K0  P62745  Q8NFJ5  P24557  P35556  P05089  Q8IUX7  Q02410  Q96AQ8  O43688  O15162  Q9HB63  Q8TAF7  Q9H825  P78316  Q5T749  Q96IJ6  Q96QE5  O95832  Q96S97  P02753  Q8IVH4  P60880  O00468  P13645  Q12805  P02774  O60487  Q8TC26  Q8TAA9  P81605  Q5VUM1  O14593  Q9NRQ2  P05783  Q86YS6  Q9BY49  P53801  Q92504  Q7Z6I8  Q14767  Q8N129  Q96CG8  Q8IVF4  Q5SR56  Q8NC42  P81408  Q8NCU8  Q13740  Q92547  Q9NYP9  Q14563  Q96CU9  Q8IUX1  P22413  P11047  P05120  O95870  Q92947  P31944  O75031  Q96LX8  Q96P63  Q5BJD5  P04004  Q8NBK3  Q9NX61  P04439  P27105  Q5BJH2  Q96EL3  Q9GZM7  P49810  P15529  Q9NWT8  Q96QD8  Q8N878  Q8WUZ0  Q02446  Q14494  P78383  Q86WW8  P06756  P10398  P16144  Q86YZ3  Q9NZH0  O14545  O15258  Q92959  Q96MU7  Q6YHK3  P35908  Q9HB58  Q86SX3  Q99442  Q9NV92  O14924  P02649  O60353  O15091  P31431  O43824  P60033  P55085  Q17RC7  Q9BQ49  O43427  Q96QA5  Q6ZN54  Q9BRT2  P15151  Q0ZGT2  Q7Z6M3  Q15035  Q9BXP2  P35237  Q13201  P17301  Q8N6G6  Q96MX0  P48307  Q7Z403  P21926  P35968  Q15434  Q96GP6  O95785  P57087  O00481  Q53TQ3  P13647  O43716  P02771  O95907  P12273  P15907  P50225  P47929  Q8IUW5  Q9H7D0  Q969Y2  Q8N8Q9  Q8WWI5  A0A0B4J2D5  Q9Y2R0  P10909  Q9UBH6  Q96C57  O00501  Q9NVS2  P25311  Q08431  Q86WC4  O95858  P29279  Q8WVV4  Q92738  Q9H9S5  P04264  P29508  Q9Y672  Q96ND0  Q9UHQ4  O00257  O00311  P85037  Q6UW56  Q9UFN0  Q9HAW4  Q9NX18  P35613  Q13243  Q53H96  Q13873  Q8IVS2  P13688  Q9BXJ4  Q9P0T7  O95980  A8MT69  P16104  Q08188  Q6ZMP0  P61604  P78410  P61020  Q6ZSR9  Q9NQG5  P10321  Q99075  Q9NZB8  O15427  O94813  P07919  Q9BRJ2  Q14956  P56817  P07942  Q9UGY1  Q9H4E5  Q9Y2G9  O43677  Q9UBG0  Q6KB66  Q14126  P57078  Q16363  O60462  Q14108  P20339  P27487  Q9BZM1  P48594  Q96K19  Q92794  P14927  Q5HYA8  P0C0L4  Q96NB2  Q9C0K1  O60613  P48509  Q96JQ2  Q9Y4A8  Q9Y5S1  Q96N46  Q6IEG0  Q9Y276  P16284  Q96QE2  Q9Y6A9  Q9Y6L7  A6NFY4  Q96HV5  P58335  Q8NB78  Q8N350  Q53F39  Q9Y4C1  Q9BU23  P54289  Q96Q45  P98172  P09622  Q6UWJ1  Q8IW75  P35555  P13497  Q8IZN3  O60292  Q9Y639  P02533  Q9NPJ6  Q9H3E2  Q9BRK5  Q9BY77  Q9NZJ5  A6NM45  Q8TCT9  Q9NUJ1  Q9HCC0  O95707  P21397  Q99549  P62079  P49247  P01034  Q9UL03  P00747  Q10588  Q8IWA5  Q8IV61  Q15043  Q99715  P24001  O75648  P35030  Q96CS7  Q6UXD5  Q96SL8  O14657  Q9NS93  O15243  O14863  P82912  Q6PK81  Q96H55  P22735  P15884  Q96A57  P22528  Q86X95  Q8NI22  Q99685  Q96C10  Q99720  Q96GZ6  Q9BZC7  Q9UHF1  Q68CZ2  P05121  O43854  O95302  P23368  P43007  Q6V0I7  Q9UQR0  P15924  Q9UHJ9  Q9Y2D5  Q9UBN6  Q96RQ3  Q9Y3A2  Q9NUM4  Q6P587  Q9UKR5  P08603  P40926  P07225  P15289  Q8IZF2  P05496  Q99574  P51690  Q15528  P00750  Q19T08  Q9NUN5  Q92750  P01024  Q9C0C4  Q9GZX9  O43674  Q9BTK6  Q01167  Q96BT3  P23786  Q9BQT9  P56557  Q6MZP7  Q9Y624  O14561  P24530  Q9NP80  Q9NX76  O14647  Q9NNW7  Q9BX97  Q6UB28  Q8TA86  P02538  Q9ULG1  Q9H930  Q9ULR0  Q9UMX1  O00322  P05362  Q9HCM1  Q8N7R7  P10646  P82909  Q8N3L3  Q7L2Z9  Q9Y2Q0  Q96EZ8  Q9H0H5  P09486  P61769  Q9P2G4  P18084  Q8NDX5  Q96CE8  Q96F15  Q9HDC9  Q71F23  P53367  P21589  P55001  Q12872  Q9H330  Q9UGM6  Q8N5G2  Q8WY22  Q9C073  O14672  Q13835  Q99653  Q9Y548  Q9UBF2  Q13133  P14324  P54136  Q9BW83  Q9NRG1  O76094  Q13503  Q04446  Q9Y3E1  Q9Y4C2  O00628  P22234  Q8N9N7  P53611  P62244  Q8NFZ0  Q05397  Q9UNH7  P24539  O75306  Q86V21  P27708  Q8WUU5  Q2M2I8  Q8N8N7  Q8IYD1  Q9H974  Q8N3C7  O43681  Q9UGN5  Q06546  P49406  Q8WUH1  Q8WVP5  Q8TB36  Q96EK5  P52306  Q9H488  Q9BTW9  Q9NR61  Q6P1J9  Q9H0U4  P24468  A0AVT1  Q8WX92  Q9C0H6  Q9Y224  O00499  Q8IXM2  Q9HA64  Q9BUI4  Q06323  Q99735  P46020  Q9H8H2  P62140  Q8WVK7  Q9Y5P6  Q8N584  P53992  Q9BVG4  Q96F85  P54619  Q6GMV2  P30046  P36405  Q86UX7  Q15181  O00258  Q86V81  Q86TI2  P78318  Q15436  Q8IY47  Q8NBT0  Q9H098  Q8N5M9  Q9BWH2  Q9P2J5  O15067  Q6NXE6  Q14C86  Q15029  Q86X52  O95671  O00203  P54819  P60228  P13984  Q5T0D9  Q9UQB8  O75436  Q969T9  P49327  Q86UK5  O60547  P48506  Q9BVI4  P14635  Q9BTV5  Q9Y281  P50749  Q9Y5K5  Q04917  Q9BZG1  Q16875  P15374  Q9Y262  P18077  Q16698  P36915  Q13347  O43768  P61221  Q9Y678  P04792  Q9H490  Q8WUX2  Q8N4Q0  P52179  P51114  Q562E7  Q99962  Q14012  P35080  O75787  Q5JS54  P37287  P61353  O00625  Q9NPD8  P19367  Q9NWV4  A6NHL2  Q06203  P25398  Q8N4P3  O15305  P49459  Q96TA1  P28161  P07437  Q15311  Q8TEV9  Q6P2E9  Q53EL6  Q9Y5X1  Q99942  Q9H4H8  P55786  O94874  Q9NNW5  Q15819  Q96HR8  P22083  P60520  P18669  O95486  Q969U7  P48643  P40967  Q9Y371  P40227  P48556  Q9NR28  P56211  P84085  Q9H3K6  Q99615  P68104  Q6NYC1  Q495W5  P61081  Q15942  Q9Y2Z4  P54652  Q96F24  Q9UH65  Q07960  Q9GZT3  P55884  P61313  Q13619  P61201  P07900  O95069  Q9UHE8  P17655  P45974  Q96KB5  Q5KU26  P08237  Q8IYB5  O00160  O00471  Q6NW34  O43805  P31948  P15927  Q9NTK5  P08133  P62249  Q9Y613  P23396  Q9UPN7  Q96FV2  P48047  P53007  P36871  Q8WVY7  P63244  Q8IXU6  P48730  Q99973  Q15185  Q76MJ5  P13489  P00338  P46779  Q9H074  B5ME19  Q99836  Q8TBC4  P26038  P62266  Q6Y7W6  O75143  P62701  Q9NR46  P52788  Q9NVI7  P23526  P53396  O43861  Q9Y2R5  Q9H492  P07992  Q5TFE4  P05386  P23528  Q86WQ0  Q92530  P04406  Q9BQ04  Q9H4M9  O95178  Q9H299  Q8IZ81  P62424  P49902  Q9UIL1  Q5TDH0  O15111  Q9NWZ8  Q9NUP9  P06737  Q8N5Y2  Q14139  Q9Y2T2  Q13614  P16949  Q9UBV7  P11766  P58546  Q9Y496  P07355  P14618  O75391  P63104  Q9NP79  Q562R1  Q03013  Q12996  P35241  Q13951  Q17RB0  O43741  O00151  Q6NZ67  Q00978  Q96HW7  Q99614  Q14847  Q13405  O00303  P20073  P22307  P09936  Q12768  Q8IYS0  Q13617  Q9P2M4  Q13395  Q9UNQ2  Q92572  P22681  Q7L1Q6  O95373  Q14CX7  O75496  Q9H832  Q99490  P68366  Q4J6C6  P52298  Q4KWH8  P62258  Q9BX68  Q9Y2S2  P56378  Q01813  Q9UQ80  P13693  Q8IUH4  Q8NB15  P51397  Q5T2R2  Q8TDQ7  O43765  Q9UDX5  P14317  Q13496  Q70Z53  P63098  P63241  Q86X27  P39019  Q9H2D1  Q00325  Q9NWT6  Q96MW5  P04424  Q9UDX4  P21399  Q9BSJ2  P51812  Q9UQR1  P46778  P61204  P55210  Q9Y2V2  Q15025  Q8IWX5  Q6QNY1  P53680  Q6P1X5  P0CW20  Q9BPX7  P49754  O95857  Q9Y221  Q99584  P47224  P61923  Q9H993  Q92522  Q6P9B9  P19623  O14641  Q5JTZ9  P63000  Q8WZA0  Q96AC1  Q147X3  Q9Y6B6  O75525  Q9H0A8  P11172  Q8TDN4  Q9NZZ3  P09211  Q8N726  P68036  Q92604  Q9UMS0  Q13227  Q15286  Q96HJ9  O00142  Q66K74  Q9NRD5  P62829  Q15814  P24534  O95166  O75935  Q14019  P50502  Q9UBI1  P09104  P61086  Q96EB1  P38571  O95372  Q15056  Q7L5D6  Q5XKP0  Q16629  Q96CB9  Q14155  O00244  P07737  P52209  Q9Y316  P49354  Q9Y5Z4  Q14376  P40429  Q9H8W4  P36959  Q9BUE0  Q08945  Q9ULI0  Q9Y4P8  Q9BVM4  Q16401  Q9H4I9  P83731  P49770  Q8NBF2  P28845  O75473  Q712K3  P51668  P62993  Q14694  Q15561  P50395  Q8TBP6  P16403  P59998  P53004  Q15382  P06733  Q93045  P27361  Q96GQ5  Q6XE24  Q9UPW0  Q86VV8  Q96IX5  Q9NRF8  Q9HC21  Q96GG9  Q96IK0  Q9NX20  Q9H3H3  Q9BTV6  P14406  O00273  Q9UBP0  Q13155  Q96T21  O15212  Q8TEB1  Q7L9L4  P05408  Q9NPJ3  Q9NX08  O43598  P52943  Q9Y3C8  Q13901  O95229  Q9NUV9  P32119  Q6P4I2  Q99426  Q07283  P46926  P30626  O75575  P49207  P62826  Q00059  P16401  Q13188  P31150  Q9UL33  P07311  P06703  Q8TE57  Q6P9F5  P31949  P62256  P63313  Q96C01  O14653  P07741  Q8WVT3  P39687  Q13011  P29558  O15347  Q92688  Q9BRT6  O95298  Q15006  Q9NRX1  Q7L592  Q8TDI8  Q8NBX0  Q15041  P09496  Q9UK99  O96033  Q9HCJ6  P61088  P17535  Q9NPD3  P62277  Q9H7E9  O00762  Q5QNW6  P62081  Q68CQ7  O60925  Q14469  Q9UL42  O00401  Q96AZ6 | TSPAN8  KRT24  SEC31B  RUBCN  DNAI7  GFRA1  KRT7  SULF1  FLVCR2  ACTRT1  IKZF3  TTLL6  SLC43A2  RIPPLY3  NDUFAF8  FN1  IGLC2  CPNE7  TMEM267  IFITM2  BDP1  GGNBP1  TGFB2  IGHA1  MXRA5  MSANTD4  JADE2  PRSS3P2  SEMG2  LYSMD3  KRT8  COL18A1  TLR9  ABI3BP  CD34  S100A7  FAS  DCN  IL4R  FLG2  COL4A1  SHH  CD200  LPAR1  DENND6B  LTBP1  DHTKD1  TUBA3D  CXCR4  NAV3  SLC39A1  C1orf53  HSP90AA4P  COL8A1  ARPC5L  S100A14  H2AZ2  KIFC3  MATN3  NBEA  RFTN2  CCHCR1  COL6A2  DES  TF  LYZ  PCLO  NSUN2  KLK11  TRIQK  KIF26B  ADAMTS1  SCYL3  CD55  IGFBP7  GVQW3  ALDH1B1  SCGB2A2  EMCN  THSD7A  LHFPL2  KRT6C  PLP2  SDC2  TRA2B  PLPP3  EMP2  LOXL2  GRAMD1B  IGKC  UNC93B1  GASK1B  SLC7A11  ITGB3  PLPP1  CCN1  S1PR1  COL5A1  APOA1  SEMA3D  MUCL1  TADA3  NID1  APOH  SRPX2  TNFRSF10B  HSPG2  TNFRSF17  SMPDL3B  SMAGP  AMBP  SCARB1  TSEN34  TM4SF1  SLC35A1  ITGAE  SLC25A27  SPOUT1  COL5A2  COL4A2  LORICRIN  TNFRSF10A  SLC15A3  SLC4A4  GINM1  TMPO  PRSS1  H2AC21  PPP1R16B  KRT78  TMLHE  FLG  ARMCX1  HGF  HPCAL4  IRF7  KDELR2  IGLL5  NCOR2  SNN  A2M  RNF121  MGARP  HMGA2  RASL10A  BCKDHB  TSPAN3  CDKN2C  DGAT1  NID2  HLA-F  LIX1L  CYBA  KRT77  RETREG3  SLCO4A1  THBS3  SPAG1  AAR2  ALKBH1  FOXP4  BLOC1S5  TMEM44  RHOB  GPRC5A  TBXAS1  FBN2  ARG1  AEBP1  APBA1  MCUR1  PLPP2  PLSCR1  NTN4  ZNF461  METTL8  NOP14  KPRP  GMPPA  TEFM  CLDN1  MYADM  RBP4  MMAA  SNAP25  AGRN  KRT10  EFEMP1  GC  MPZL2  TMEM163  VANGL1  DCD  SDHAF4  RFXANK  PLSCR4  KRT18  RAB43  PECR  PTTG1IP  SLC39A7  C5orf24  LTBP2  CNPY4  CTHRC1  DNAH10  MFSD14B  RNF149  ENTREP3  MTLN  ALCAM  TOPBP1  MIS18A  SEMA3A  FOXRED1  TMEM126B  ENPP1  LAMC1  SERPINB2  ABHD16A  GCDH  CASP14  HSF2BP  ZNF597  SERPINB12  TMEM41B  VTN  SUMF1  TMEM161A  HLA-A  STOM  TMEM128  MRPL53  TINAGL1  PSEN2  CD46  AURKAIP1  SLC38A2  FRMD1  BCL7C  SP4  NFE2L1  SLC35B1  COA5  ITGAV  ARAF  ITGB4  HRNR  GPRC5B  TRAFD1  RER1  SLCO2A1  YTHDC1  CD109  KRT2  SP110  TEDC1  SEC62  NDFIP2  RGS12  APOE  FZD6  PRORP  SDC4  GTPBP6  CD81  F2RL1  EXOC3L4  SMIM7  FIBP  GSDMA  DEF8  UQCC2  PVR  NEXN  MILR1  TRAM2  SLC12A9  SERPINB6  MMRN1  ITGA2  ADAMTSL1  CMTM3  TFPI2  TMC6  CD9  KDR  RBMS2  SCARF2  WIZ  JAM2  BTN3A1  INO80D  KRT5  GATC  AFP  SLC16A8  PIP  ST6GAL1  SULT1A1  LGALS7B  RELL1  DOCK5  GTPBP3  NIPA2  SLC44A1  GATD3B  COA3  CLU  XPR1  CUSTOS  CLDN5  MRPS18A  AZGP1  MFGE8  OSTM1  TSPAN15  CCN2  POF1B  USP6NL  FKRP  KRT1  SERPINB3  ALG6  FAM210A  BCAP29  CBX4  CDC7  FOXK1  ATRAID  NIPSNAP3A  CLSPN  SDHAF2  BSG  SRSF5  PYCR3  BMPR2  MCAT  CEACAM1  C1QTNF3  TMEM9  RECK  CENPX  H2AX  TGM3  THSD4  HSPE1  BTN3A2  RAB5B  RPRD1B  HLA-C  HBEGF  MOCS1  SLC16A3  SLIT2  UQCRH  MRPL45  GPNMB  BACE1  LAMB1  NOL12  RHOJ  SBNO2  NDUFC1  MRC2  KRT80  DSG2  RIPK4  LAMA4  NRP2  SCARB2  RAB5A  DPP4  PLA2G12A  SERPINB4  RNF170  KAT6A  UQCRB  TMEM67  C4A  SFXN2  SLC39A8  SELENOF  CD151  CLMN  NFE2L3  TRPV2  TTC14  SNRNP48  BCS1L  PECAM1  SLC2A13  SPCS1  TLL2  NEMP2  TMEM41A  ANTXR2  KDM1B  CBARP  MPPE1  KDM3A  LMF2  CACNA2D1  TMEM237  EFNB1  DLD  TMCO3  SERPINA12  FBN1  BMP1  ZDHHC14  SIPA1L3  NPTN  KRT14  MED4  SNX25  SDF4  POLDIP3  EIF2AK3  CLDN24  HM13  ABHD10  MCCC2  POP4  MAOA  MPHOSPH8  TSPAN5  RPIA  CST3  INTS6  PLG  BST1  SLC44A2  RASGRP3  SLC39A14  COL12A1  IL32  TRMU  PRSS3  PLEKHB2  SEZ6L2  FIZ1  TOR1B  TM7SF3  LEPROT  SLC30A4  MRPS11  ZNF773  MYO19  TGM1  TCF4  TMEM230  SPRR1B  CIR1  MCFD2  MGLL  DHX58  SIGMAR1  SLC41A3  ABCA2  EGFL7  TNS3  SERPINE1  EDIL3  FKBP9  ME2  SLC1A4  FAT4  SCML2  DSP  PGAP2  AKAP2  TNFRSF10D  MCCC1  UTP11  TMEM106B  FAHD1  ERG28  CFH  MDH2  PROS1  ARSA  ADGRF5  ATP5MC1  SERPINI1  ARSL  MED22  PLAT  ECSCR  LMBRD1  TAF4B  C3  SEMA4C  TWSG1  NDUFB5  PAGR1  FOXK2  CENPT  CPT2  CLSTN3  TMEM50B  LIN54  F11R  NDUFAB1  EDNRB  PNPLA8  CMTM6  CHD2  TXNRD2  PLVAP  METAP1D  RP9  KRT6A  INO80  SP140L  ISY1  SUFU  UPK1A  ICAM1  RESF1  CCNYL1  TFPI  MRPS36  TXLNB  CENPQ  ATP8A1  MCRS1  RACGAP1  SPARC  B2M  MAP10  ITGB5  PHC3  TM4SF18  GIMAP5  APMAP  CENPU  ARFIP1  NT5E  MFAP2  SFSWAP  TMEM245  WARS2  MACO1  BRI3BP  FAM117A  ADAM10  PKP1  CHP1  YIPF1  COPG2  NR1H3  FDPS  RARS1  IFT27  PRTFDC1  SRP72  MED21  GBE1  HDGFL3  TCAF1  PEX7  PAICS  LRRC57  RABGGTB  RPS15A  FBH1  PTK2  SNX6  ATP5PB  NDUFS2  AACS  CAD  GATAD1  AAK1  PTGR2  GSPT2  QTRT2  CLIP4  GET3  PARP2  GABPA  MRPL19  CHURC1  TNFAIP8L1  GDAP1  KIFBP  RAP1GDS1  POFUT1  TBCD  DLL4  CDC73  RAB1B  NR2F2  UBA6  NELFB  KLHL4  RTRAF  BIN1  BAP18  FN3KRP  POLR3C  PSME1  MGST2  PHKA1  DDX31  PPP1CB  SKA2  GMPPB  TTC39C  SEC24C  PBDC1  CNRIP1  PRKAG1  SMYD5  DDT  ARL3  FERMT3  PPA1  GET1  ALYREF  DPP9  IGBP1  SEC23A  KBTBD2  POC1A  FAM107B  JAGN1  FUNDC2  LARS1  PFAS  ARMC6  GAPVD1  EFTUD2  CHSY1  ASMTL  AP3B1  AK2  EIF3E  GTF2F2  TPRG1L  BAIAP2  VPS26A  WBP2  FASN  EVC2  GMDS  GCLC  NOC4L  CCNB1  FSD1  CFL2  RASSF2  UCHL5  YWHAH  RAB34  PFKFB3  UCHL3  EIF3L  RPL35A  DECR1  GNL1  EIF3I  ENSA  ABCE1  COPG1  HSPB1  PIGU  CHAC2  PTGR3  MYOM1  FXR1  WDR81  SH3GL2  CAMK1  PFN2  ATP6AP2  PSMG4  PIGA  RPL27  PIR  UBE2T  HK1  CZIB  TUBAL3  PPAT  RPS12  HDDC3  PMM2  UBE2A  NIBAN2  GSTM2  TUBB  RALBP1  SMCR8  EDC4  PDCD4  SNX9  RNF5  FAM83D  NPEPPS  UFL1  WDR6  UBE2V2  NAF1  FUT4  GABARAPL2  PGAM1  SEC24A  PSMG2  CCT5  PMEL  SH3GLB1  CCT6A  PSMD8  DIABLO  ARPP19  ARF5  BOLA2B  DNAJC7  EEF1A1  JMJD6  FUT11  UBE2M  ZYX  YARS2  HSPA2  NRBF2  SWAP70  ARHGAP1  SLIRP  EIF3B  RPL15  CUL4A  COPS2  HSP90AA1  KCNK2  STEAP1  CAPN2  USP5  PBK  COLEC12  PFKM  SMAP1  MYO1F  EXOC5  NEPRO  SSNA1  STIP1  RPA2  OLA1  ANXA6  RPS16  FHOD1  RPS3  PPP6R1  SCRN2  ATP5PO  SLC25A1  PGM1  UBLCP1  RACK1  SLC35F2  CSNK1D  TEP1  PTGES3  ERN2  RNH1  LDHA  RPL28  PAIP1  EIF3CL  MYD88  UBA3  MSN  RPS23  GIGYF2  ATG13  RPS4X  SH3GLB2  SMS  ATAD3A  AHCY  ACLY  ATP9B  MRPS17  MAP1LC3A  ERCC1  NT5DC1  RPLP1  CFL1  NR2C2AP  PSMF1  GAPDH  RBM4B  EHD1  NDUFB2  SH3BGRL3  ELMOD2  RPL7A  NT5C2  SCOC  DDI2  CHUK  GEMIN8  LIN7C  PYGL  MSL3  UBE4A  AP3M1  MTMR2  STMN1  B4GALT7  ADH5  MTPN  KIF3A  ANXA2  PKM  SPAG7  YWHAZ  VTA1  ACTBL2  GSTM4  CSTF3  RDX  CBFB  RTL8B  PRKAB2  PDLIM1  MZT2B  IRF9  INTS4  TTC1  LASP1  MRPL49  EIF3F  ANXA7  SCP2  UCHL1  WASHC5  GRAMD1C  CUL2  TBC1D14  TARBP1  DIMT1  AP3S1  CBL  BZW1  IPO7  NAA25  GMNN  UBE2Z  AGAP2  TUBA4A  PREPL  NCBP2  PLCH1  YWHAE  HINT2  CRYL1  ATP5MJ  PFKP  PA2G4  TPT1  ZDHHC13  ZNF511  DAP  PDSS1  GNPDA2  SGTA  MTFP1  HCLS1  MTM1  FRA10AC1  PPP3R1  EIF5A  RALGPS2  RPS19  SLC25A32  SLC25A3  HIF1AN  COG8  ASL  SEC14L3  ACO1  TUBGCP2  RPS6KA3  ZNF148  RPL21  ARF3  CASP7  CARHSP1  TNIP1  SGPP2  BLOC1S2  AP2S1  TAF2  LIMS4  C7orf25  VPS41  TSPAN13  NIP7  S100A13  RABIF  COPZ1  ARMT1  H1-10  INTS5  SRM  DVL2  AARS2  RAC1  LZIC  FERMT2  NAA30  SAR1B  KHDRBS3  COMMD4  UMPS  CABLES1  CHMP5  GSTP1  CDKN2A  UBE2L3  LPGAT1  NFU1  GPS2  RAB35  FMC1  TK2  MAP1S  PICK1  RPL23  TBCC  EEF1B2  GABARAP  DCTN3  COTL1  ST13  COMMD3  ENO2  UBE2K  ELP4  LIPA  LYPLA2  EIF4H  GET4  MICOS13  SRSF7  NSUN4  ARHGEF7  ATOX1  PFN1  PGD  MEMO1  FNTA  HEBP2  GALE  RPL13A  PLEKHF2  GMPR  MED18  SSRP1  ATAD2B  WIPI2  GGACT  PSMD5  SMDT1  RPL24  EIF2B2  NHLRC2  HSD11B1  LGR5  UBE2R2  UBE2D1  GRB2  USP10  TEAD4  GDI2  SLC25A40  H1-2  ARPC4  BLVRA  RHEB  ENO1  STMN2  MAPK3  RUSF1  RBMS3  FOXJ3  RTTN  ATP5MK  CTPS2  SLC25A19  DCUN1D1  TMEM101  MRPL16  C11orf68  DPH7  COX7A2  DFFA  SPAST  AIMP2  SECISBP2  PFDN6  DCAF11  MOB1B  SCG5  ACOT13  COMMD8  DNPH1  CRIP2  UFC1  C1D  ZWINT  GIMAP4  PRDX2  WDR73  TBCB  TCHH  GNPDA1  SRI  CRCP  RPL34  RAN  TFAM  H1-5  STK3  GDI1  TRAPPC2L  ACYP1  S100A6  ADAMTS16  TRIM40  S100A11  UBE2H  TMSB10  FAM136A  GOSR2  APRT  TRAPPC12  ANP32A  ECH1  RBMS1  HMGB3  ANP32B  LLPH  NDUFC2  EMC2  PNO1  NDUFAF7  TMC1  SCCPDH  ARL6IP1  CLTA  FBXO3  MOCS2  VAT1L  UBE2N  JUND  EXOSC4  RPS13  C8orf33  UBE2C  H2BC18  RPS7  GLT8D1  PFDN1  HES1  PNMA2  WASL  ISG20 | 26  55.1  128.6  108.6  83.1  51.4  51.4  101  57.2  41.7  58  96.3  62.7  20.4  7.8  272.2  11.3  70.2  24.2  14.6  293.7  12.3  47.7  37.6  312  41.1  87.4  26.5  65.4  34.5  53.7  178.1  115.8  117.8  40.7  11.5  37.7  39.7  89.6  247.9  160.5  49.6  31.2  41.1  66.4  186.7  103  49.9  39.7  255.5  34.2  15.5  47.7  73.3  16.9  11.7  13.5  92.7  52.8  327.6  55.9  88.6  108.5  53.5  77  16.5  560.4  86.4  31  9.7  223.7  105.3  82.8  41.4  29.1  29.4  57.2  10.5  27.4  185.2  24.5  60  16.7  22.1  33.6  35.1  19.2  86.7  85.3  11.8  66.6  57.5  55.4  87  32.1  42  42.8  183.4  30.8  89.6  9  48.9  136.3  38.3  52.9  47.8  468.5  20.2  50.8  10.7  39  60.8  33.6  21.6  36.8  130.1  36  42  144.8  167.4  25.7  50.1  63.5  121.4  36.8  50.6  26.5  14  63.5  56.8  49.5  434.9  49.2  83.1  22.2  54.2  24.4  23  273.5  9.5  163.2  37.9  25.4  11.8  22.5  43.1  28  18.1  55.2  151.2  39  36.5  21  61.9  51.4  77.1  104.1  103.6  43.4  43.8  73.4  21.6  52.2  22.1  40.2  60.5  314.6  34.7  130.8  92.8  39.7  32.6  35  70  66.2  33.4  97.6  64.1  46.3  41.7  22.7  35.3  23  46.5  23.3  217.2  58.8  54.6  52.9  24.5  31.4  59.9  11.3  12.2  28.1  37  48  23.3  32.5  20.3  50.1  20.1  194.9  28.3  26.2  514.5  54.5  43.1  71.3  6.5  65.1  170.6  25.8  88.8  53.8  25.9  104.9  177.5  46.6  63.2  48.1  27.7  37.6  48  46.2  32.5  54.3  40.5  53.6  40.8  31.7  18.8  12.1  52.4  50.1  43.7  22.3  56  62.5  23.5  81.9  84.7  35.7  8.4  116  67.5  202  282.2  44.8  64.8  22.9  70  84.6  161.6  65.4  78.3  54.2  45.8  36.4  156.3  36.1  79.2  67.3  21.6  56.9  25.8  44.1  79.8  8.6  41.9  49.3  58.7  14.9  45.3  80.6  38.7  43.3  96  42.6  138  129.2  193.3  19.7  26.9  90  25.4  151.4  43.9  92.3  178.6  33.2  57.6  113.1  62.3  15.1  68.6  52.3  16.6  46.6  34.1  15.1  29.3  215.2  52  39.2  73.3  28.1  11.7  52.5  81.5  28.2  23.1  22.2  34.2  43.1  37.2  33.1  38.1  68  94  54.5  66  44.5  58.1  30.8  28.3  61.3  63.8  75.4  24.7  28.4  151  19.6  42.2  31.2  28.6  115.1  42.9  57.5  27  20.6  106.4  9  15.1  76.6  112.4  10.9  36.4  23.7  38  36.9  40.6  23.1  70.1  49.4  169.8  10.7  35.3  63.9  55.7  197.9  24.6  23.8  150.2  8.7  166.6  50.5  122.2  91.6  202.4  104.8  54.3  23.6  88.2  21.1  44.8  29.8  224.9  13.5  111.7  192.7  36.2  49.6  18.1  28.3  111.6  76.1  85.9  88.3  39.9  47.5  82.5  70.3  18.3  113.5  48.6  29.6  53.6  92  73.9  45.1  147.2  79.6  124.5  45.5  38  54.1  75.5  47.1  312.1  111.2  53.4  194.5  44.4  51.5  29.7  97.9  41.8  46.1  125.1  24.4  41.5  33.9  61.3  25.4  59.6  97.1  30.3  33.2  15.8  100.3  90.5  35.7  80.1  78.3  54.2  332.9  26.7  47.7  32.5  24.7  97.5  52  38  64.1  14.2  47.5  20.6  50.5  109.1  89.7  71.3  13.2  9.9  52.3  16.4  33.2  76.6  25.1  54.7  269.7  29.6  155.2  45  53.7  63  65.4  55.7  542.4  77.2  331.6  29.4  94.6  41.8  80.4  30.4  31.1  24.8  15.9  139  35.5  75.1  53.6  149.4  14.3  46.4  65.6  22.2  62.9  21.3  61.3  91  187  92.6  25  21.7  27.7  69  60.4  73.7  106  17.9  79.4  32.6  17.4  49.6  88.4  20.4  211.2  56.5  50.6  37.1  26.1  60  176.6  67  33  53.9  28.9  57.8  194.7  40.7  35  11.5  76.5  30.6  131.3  51.8  71  34.6  13.7  100.3  88  106.1  22.3  34.8  46.5  47.5  41.7  63.3  20.8  104.8  97.3  40.1  76.1  27.8  48.3  84.1  82.8  22.4  34.3  97.6  50.4  48.2  75.3  20.5  25.7  74.6  15.6  80.4  22.6  102.1  35.9  47  26.7  36.9  14.8  117.6  119.2  46.6  28.9  52.5  75.1  242.8  28.7  103.8  38.5  68.8  46.7  76.3  38.8  66.2  51.3  33.5  12.9  20.8  41.3  71.8  66.3  43.9  132.5  74.6  60.5  22.2  45.5  117.9  65.7  80.2  28.1  64.7  17.9  34.4  60.6  28.7  16.6  137.2  94  37.2  14.2  39.8  65.8  118.2  26  18.6  37.6  47.3  12.7  20.4  75.9  32.6  19.8  26.9  98.2  39.2  86.1  71.3  45  15.5  21.1  20.7  134.4  144.6  54.1  164.9  109.4  91.7  68.8  121.2  26.5  52.2  28.4  30.2  60.8  38.1  28.1  273.3  147.9  41.9  72.7  58.4  48.3  55.8  18.7  37.8  37.6  28.2  29  59.6  26.2  66.7  12.5  36  68.6  36.5  13.4  67.3  97.7  22.8  50  20.9  40.1  187.5  69.7  211.6  39.9  41.3  15  39  13.8  54.1  15.8  32.1  22.5  102.4  18  49.9  57.4  14.5  20.3  28.1  17.3  84.1  25.7  49.6  76  105  151.6  51.7  66.6  19.9  64.4  103.2  89.5  121.6  16.4  53.7  59  13.7  28.8  119.7  29.4  59.6  70.2  40.8  58  39.6  27.1  12.3  20.5  10.1  56.4  50.1  46.4  55.8  20.9  61.2  53.2  70  32.4  69  50.4  12.3  92.4  24.1  87.6  51.6  84.6  47.1  39.8  79.9  95.7  36.1  81.5  85.1  50.4  124.8  81.8  64.5  13.6  62.6  29.2  44.7  75.8  16.4  126.5  26.7  96.7  46.6  23.3  34  61.4  36.8  35.1  41.2  47.3  290.3  18.7  102.4  49.9  36.7  15.7  53.5  105.4  33.2  51.8  67.8  15.8  150  56.5  29.6  43.9  41.2  71.3  47.7  120.8  129.2  14.5  14.3  32.5  51.8  11.5  18.5  15.9  29.8  36  40.1  60.6  12.1  10.4  34.9  30  64.9  18  44.5  84.6  28.6  21.8  97.1  59.8  122.5  46.9  73.3  17.3  37.4  39.7  12.9  80  38.6  57.9  26  27.7  33.9  42  25.5  82.9  68.5  21.5  13.2  30.3  36  16.2  43.7  108.1  33.5  29.7  19.2  37.5  52.7  59  24.8  134.2  76  86.9  78.1  181.6  35.2  21.7  99.6  48  119.4  112.2  23.6  38.2  124.6  49.9  83.9  18  189.1  29.2  17.2  35.4  6.7  85.5  43.8  19.6  70.8  28.2  11.2  46.2  31.1  34  18  54  69.9  37.5  19.3  16.8  65.1  16.1  35.4  40.1  40.3  68.4  51.6  46  98.3  102.5  83.7  88.9  18.6  20.6  34.3  15.9  71.8  44.7  16  17  136.9  13.2  46.4  98.5  22.1  20.4  11.5  13.8  20.2  51.1  22.5  107.9  33.8  78.9  107.3  21.4  21.5  77.8  39.3  22.4  38.8  21.8  52.2  67.6  24.6  23.3  13.9  17.9  43.1  28.4  36.7  23  12.7  31  112.1  46.6  14.9  39.2  24.7  13.9  21.1  15.9  41.3  22.1  47.2  22.4  46.6  45.4  24.7  27.4  36.5  13.1  27.4  43.1  90  7.4  15  53.1  33.7  44.4  22.9  38.3  23.6  27.8  37.4  23.6  81  164.8  49.4  17.3  56.2  11.4  17.8  39  79.4  32.4  99.9  27.1  16.6  25.2  87.1  48.3  50.6  38.1  21.4  19.7  33.4  20.5  47.1  20.8  43.1  51  47.8  68.9  248.5  6.5  65.6  35.5  30.1  28.8  28.4  31.4  50.5  9.4  36.5  67.2  35.3  95.4  14.6  61.6  25.1  23.7  15  21.1  19.1  22.5  19.4  16  31.3  37.5  21.9  41.7  27.3  253.8  32.6  21.7  16.9  13.3  24.4  29.1  22.6  56.3  50.6  16.1  11.3  10.2  136.1  29.3  11.7  20.6  5  15.6  24.8  19.6  79.3  28.6  35.8  44.5  23  28.8  15.2  14.2  34.8  27.9  49.2  87.7  47.1  23.3  27.1  54.5  9.7  45.9  17.1  35.2  26.4  17.2  25  19.6  13.9  22.1  41.9  14.2  29.5  41.5  54.8  20.4 | -2.63231  -2.083763333  -1.88743  -1.788256667  -1.69373  -1.68002  -1.662493333  -1.471436667  -1.385316667  -1.300113333  -1.287273333  -1.280863333  -1.24801  -1.246706667  -1.242046667  -1.23935  -1.23492  -1.229613333  -1.229313333  -1.21578  -1.21453  -1.188313333  -1.184783333  -1.18014  -1.176686667  -1.13332  -1.133013333  -1.130966667  -1.12209  -1.116076667  -1.107416667  -1.09521  -1.09079  -1.07843  -1.06684  -1.060723333  -1.05983  -1.0435  -1.0428  -1.04136  -1.041096667  -1.025156667  -1.02359  -1.021403333  -1.014363333  -1.006866667  -1.002273333  -0.97762  -0.974766667  -0.969063333  -0.957266667  -0.945083333  -0.944253333  -0.941386667  -0.93845  -0.938063333  -0.937756667  -0.93736  -0.933956667  -0.93268  -0.93242  -0.928306667  -0.9262  -0.924543333  -0.92421  -0.920793333  -0.9202  -0.919956667  -0.91907  -0.916653333  -0.915816667  -0.914423333  -0.91251  -0.911526667  -0.90646  -0.905563333  -0.90353  -0.903016667  -0.901633333  -0.89828  -0.898066667  -0.88939  -0.88834  -0.887396667  -0.886823333  -0.874286667  -0.86974  -0.86749  -0.8628  -0.85792  -0.85689  -0.850993333  -0.846083333  -0.84567  -0.84488  -0.842973333  -0.839723333  -0.83581  -0.835306667  -0.834913333  -0.828126667  -0.82793  -0.82696  -0.824326667  -0.821963333  -0.820196667  -0.81994  -0.81803  -0.814623333  -0.814333333  -0.812126667  -0.809626667  -0.80573  -0.804176667  -0.800743333  -0.800336667  -0.796853333  -0.794623333  -0.79094  -0.788706667  -0.78791  -0.78726  -0.784476667  -0.784473333  -0.784433333  -0.784343333  -0.784263333  -0.78085  -0.780603333  -0.779243333  -0.777046667  -0.775736667  -0.774026667  -0.774006667  -0.772556667  -0.770743333  -0.768473333  -0.766213333  -0.765676667  -0.763463333  -0.760293333  -0.759343333  -0.75827  -0.757026667  -0.75664  -0.75598  -0.75372  -0.75291  -0.75114  -0.74684  -0.74605  -0.74379  -0.743273333  -0.739133333  -0.738726667  -0.73866  -0.73808  -0.738026667  -0.737223333  -0.73718  -0.73611  -0.735746667  -0.735436667  -0.735433333  -0.7332  -0.732923333  -0.73217  -0.731483333  -0.731236667  -0.72995  -0.729866667  -0.729053333  -0.72904  -0.72903  -0.72889  -0.728386667  -0.72794  -0.727413333  -0.72467  -0.721446667  -0.719986667  -0.719943333  -0.716976667  -0.71633  -0.715803333  -0.714773333  -0.71306  -0.712916667  -0.712906667  -0.712326667  -0.712006667  -0.710166667  -0.709646667  -0.70857  -0.70816  -0.70774  -0.7075  -0.70661  -0.70464  -0.70437  -0.703803333  -0.701653333  -0.701406667  -0.701206667  -0.69933  -0.69842  -0.698166667  -0.697326667  -0.696983333  -0.696213333  -0.69434  -0.694076667  -0.693943333  -0.69372  -0.692886667  -0.69217  -0.692123333  -0.69205  -0.69102  -0.690753333  -0.689286667  -0.688656667  -0.687913333  -0.68745  -0.686976667  -0.68616  -0.685743333  -0.68386  -0.68085  -0.680476667  -0.679253333  -0.673946667  -0.673093333  -0.67287  -0.672683333  -0.670623333  -0.66837  -0.6678  -0.667763333  -0.665886667  -0.664763333  -0.66445  -0.663733333  -0.661386667  -0.661363333  -0.660896667  -0.660126667  -0.659116667  -0.65751  -0.657456667  -0.65745  -0.656006667  -0.655456667  -0.65523  -0.65433  -0.654136667  -0.653726667  -0.653623333  -0.653173333  -0.65281  -0.652496667  -0.6521  -0.65202  -0.65143  -0.650873333  -0.650026667  -0.64937  -0.648923333  -0.645073333  -0.64391  -0.643446667  -0.643036667  -0.64229  -0.641406667  -0.639093333  -0.63871  -0.637706667  -0.637496667  -0.63714  -0.636806667  -0.636476667  -0.63607  -0.63603  -0.634896667  -0.634116667  -0.63398  -0.633943333  -0.632616667  -0.631986667  -0.63197  -0.629746667  -0.629296667  -0.62919  -0.628593333  -0.62696  -0.62694  -0.626613333  -0.625436667  -0.625383333  -0.625346667  -0.625153333  -0.624503333  -0.62348  -0.623413333  -0.62279  -0.61786  -0.617653333  -0.61758  -0.617546667  -0.61737  -0.61593  -0.615716667  -0.615706667  -0.614886667  -0.614883333  -0.61471  -0.614343333  -0.613923333  -0.61391  -0.61361  -0.611913333  -0.610993333  -0.610846667  -0.6064  -0.606096667  -0.606063333  -0.605756667  -0.60501  -0.6049  -0.604096667  -0.603453333  -0.603113333  -0.601073333  -0.600343333  -0.599916667  -0.59979  -0.59949  -0.598626667  -0.598356667  -0.597556667  -0.597346667  -0.595466667  -0.59465  -0.593753333  -0.593443333  -0.593406667  -0.59271  -0.59257  -0.592256667  -0.591856667  -0.59171  -0.5897  -0.58699  -0.586396667  -0.58561  -0.584946667  -0.5849  -0.584266667  -0.583956667  -0.583933333  -0.582523333  -0.582426667  -0.581316667  -0.581013333  -0.580946667  -0.580723333  -0.58036  -0.57998  -0.57936  -0.579033333  -0.57867  -0.578323333  -0.577623333  -0.577503333  -0.577473333  -0.577156667  -0.576953333  -0.576293333  -0.576136667  -0.57474  -0.57423  -0.57413  -0.574053333  -0.573236667  -0.57318  -0.572843333  -0.572843333  -0.571666667  -0.571153333  -0.57115  -0.570906667  -0.57083  -0.570073333  -0.569566667  -0.567936667  -0.567773333  -0.56757  -0.566213333  -0.565276667  -0.564963333  -0.564946667  -0.564743333  -0.563606667  -0.56281  -0.562343333  -0.56222  -0.561916667  -0.561563333  -0.561286667  -0.560656667  -0.560256667  -0.559536667  -0.55919  -0.558533333  -0.558273333  -0.558113333  -0.557813333  -0.557193333  -0.55626  -0.555693333  -0.555363333  -0.555326667  -0.55505  -0.553553333  -0.55339  -0.55328  -0.552106667  -0.55195  -0.551666667  -0.55086  -0.54981  -0.549353333  -0.54924  -0.549196667  -0.54886  -0.548816667  -0.5483  -0.547716667  -0.546973333  -0.546793333  -0.546463333  -0.545826667  -0.5449  -0.544233333  -0.544216667  -0.543396667  -0.54303  -0.54303  -0.54245  -0.542023333  -0.541606667  -0.54104  -0.540916667  -0.540543333  -0.53964  -0.539363333  -0.53924  -0.539213333  -0.53891  -0.538856667  -0.53848  -0.537903333  -0.53767  -0.537623333  -0.536843333  -0.536813333  -0.53649  -0.535086667  -0.53484  -0.53246  -0.531686667  -0.53145  -0.531386667  -0.531043333  -0.53097  -0.53095  -0.530883333  -0.530846667  -0.53064  -0.53059  -0.530546667  -0.530096667  -0.52993  -0.529803333  -0.529276667  -0.529166667  -0.528773333  -0.528713333  -0.52707  -0.52689  -0.526303333  -0.525856667  -0.525666667  -0.525603333  -0.525316667  -0.525116667  -0.524586667  -0.52428  -0.524096667  -0.523466667  -0.52319  -0.522976667  -0.522773333  -0.52274  -0.522706667  -0.522253333  -0.52206  -0.521826667  -0.521436667  -0.52119  -0.521083333  -0.520636667  -0.52053  -0.51969  -0.51959  -0.519586667  -0.51909  -0.518406667  -0.518346667  -0.517463333  -0.51652  -0.51607  -0.514666667  -0.513696667  -0.513426667  -0.51302  -0.51285  -0.512013333  -0.511663333  -0.511453333  -0.51026  -0.510166667  -0.50918  -0.508583333  -0.50746  -0.506846667  -0.506666667  -0.505613333  -0.505416667  -0.504156667  -0.503303333  -0.502866667  -0.502713333  -0.502593333  -0.50214  -0.50118  -0.500916667  -0.50074  -0.50072  -0.500486667  -0.50018  0.500903333  0.501383333  0.50195  0.502773333  0.50329  0.503653333  0.503706667  0.50384  0.503916667  0.504016667  0.504216667  0.504416667  0.505236667  0.50546  0.50596  0.50599  0.506916667  0.507463333  0.507636667  0.50845  0.508616667  0.508623333  0.509096667  0.509116667  0.509796667  0.509816667  0.509953333  0.50997  0.510243333  0.510416667  0.51091  0.511296667  0.51167  0.512423333  0.512823333  0.514073333  0.514236667  0.516016667  0.516053333  0.516646667  0.51679  0.516996667  0.517133333  0.51746  0.517636667  0.517766667  0.51798  0.518776667  0.51927  0.520346667  0.520486667  0.52078  0.5212  0.521226667  0.521613333  0.522536667  0.52438  0.524823333  0.525586667  0.52581  0.52877  0.528776667  0.52928  0.529323333  0.529486667  0.530463333  0.531726667  0.5324  0.53259  0.534026667  0.53468  0.53478  0.535076667  0.535473333  0.535643333  0.536553333  0.53658  0.53696  0.537383333  0.53861  0.53973  0.54003  0.54161  0.54199  0.542326667  0.542423333  0.542506667  0.543336667  0.543576667  0.54447  0.544833333  0.545013333  0.545686667  0.546356667  0.54688  0.54725  0.54773  0.548023333  0.54886  0.54893  0.549113333  0.550443333  0.550603333  0.55082  0.55112  0.551346667  0.551846667  0.55307  0.553253333  0.554316667  0.5544  0.554726667  0.554993333  0.555183333  0.555573333  0.55615  0.55668  0.557663333  0.559646667  0.559706667  0.560426667  0.560496667  0.560543333  0.56078  0.560856667  0.56108  0.561296667  0.56141  0.561493333  0.562456667  0.562873333  0.56294  0.562946667  0.56305  0.564566667  0.56505  0.5658  0.56751  0.567866667  0.568103333  0.56819  0.568433333  0.571346667  0.572483333  0.572616667  0.572666667  0.572783333  0.573053333  0.573436667  0.57353  0.574246667  0.57465  0.575206667  0.57536  0.57575  0.576133333  0.57659  0.576646667  0.57707  0.577173333  0.57726  0.577276667  0.57771  0.578096667  0.57839  0.580086667  0.580186667  0.580506667  0.581236667  0.581426667  0.58152  0.58159  0.583193333  0.58353  0.583706667  0.583873333  0.584466667  0.585216667  0.585716667  0.586713333  0.58756  0.58857  0.589186667  0.589293333  0.589396667  0.590726667  0.5911  0.592643333  0.594933333  0.595633333  0.595763333  0.596953333  0.59709  0.5976  0.597693333  0.59778  0.598046667  0.598093333  0.59889  0.599016667  0.599053333  0.599713333  0.600053333  0.600423333  0.6005  0.601186667  0.601206667  0.601333333  0.601516667  0.603596667  0.604113333  0.604156667  0.604526667  0.605213333  0.605753333  0.606043333  0.606796667  0.607306667  0.607436667  0.607653333  0.60851  0.609063333  0.61062  0.610633333  0.613256667  0.61343  0.613536667  0.614313333  0.6144  0.615453333  0.616833333  0.61742  0.618113333  0.61852  0.619056667  0.619106667  0.61937  0.61939  0.620176667  0.623743333  0.62434  0.624783333  0.625976667  0.627416667  0.62747  0.628103333  0.63169  0.632423333  0.633293333  0.634256667  0.635986667  0.636306667  0.636663333  0.63675  0.63738  0.638386667  0.63889  0.640463333  0.641086667  0.641976667  0.642886667  0.645563333  0.646656667  0.647466667  0.649873333  0.650043333  0.651266667  0.651646667  0.652626667  0.653756667  0.65466  0.65495  0.65663  0.65787  0.65871  0.66064  0.660833333  0.66148  0.661876667  0.662106667  0.66223  0.663003333  0.663446667  0.66526  0.666166667  0.666506667  0.667376667  0.66748  0.667586667  0.668166667  0.66818  0.6683  0.66969  0.669726667  0.670596667  0.671423333  0.67399  0.675903333  0.676353333  0.676803333  0.677286667  0.677553333  0.678186667  0.678756667  0.678876667  0.68034  0.6804  0.681273333  0.681446667  0.68154  0.68182  0.68316  0.68474  0.684826667  0.684903333  0.68502  0.687386667  0.68853  0.6917  0.694663333  0.694986667  0.695183333  0.695283333  0.696883333  0.696893333  0.69827  0.69921  0.701253333  0.702083333  0.70221  0.704043333  0.70578  0.70613  0.706856667  0.707216667  0.708913333  0.71018  0.711173333  0.71967  0.720206667  0.72141  0.722216667  0.723753333  0.728333333  0.72893  0.73105  0.731323333  0.733016667  0.73493  0.735886667  0.735896667  0.736123333  0.736356667  0.737186667  0.739893333  0.740496667  0.741433333  0.74177  0.742893333  0.743146667  0.74321  0.743256667  0.745283333  0.74627  0.74795  0.74987  0.750136667  0.752316667  0.753113333  0.753313333  0.75425  0.755816667  0.7559  0.759113333  0.76247  0.763746667  0.763856667  0.763933333  0.76461  0.765466667  0.767216667  0.767943333  0.768133333  0.770776667  0.771126667  0.771316667  0.7756  0.779933333  0.78038  0.78442  0.784766667  0.785036667  0.785586667  0.78899  0.79297  0.793453333  0.795276667  0.797363333  0.798246667  0.79895  0.80057  0.80118  0.80264  0.80482  0.80487  0.804983333  0.808343333  0.8085  0.814436667  0.81451  0.81786  0.81859  0.819153333  0.820783333  0.826006667  0.831143333  0.831243333  0.83281  0.83281  0.832863333  0.8351  0.837363333  0.8382  0.839213333  0.842933333  0.843853333  0.844886667  0.849043333  0.84934  0.850426667  0.854176667  0.854776667  0.855263333  0.85572  0.861506667  0.862013333  0.863  0.8642  0.865093333  0.865223333  0.86741  0.87337  0.87875  0.883723333  0.883736667  0.893906667  0.896693333  0.896753333  0.901486667  0.902556667  0.904636667  0.905  0.90963  0.910126667  0.910186667  0.91179  0.914766667  0.919873333  0.92062  0.92138  0.922403333  0.923616667  0.92447  0.93145  0.934343333  0.93593  0.93835  0.938756667  0.948246667  0.948363333  0.951126667  0.95621  0.967623333  0.97489  0.978833333  0.990176667  0.993586667  1.001493333  1.001836667  1.003313333  1.011003333  1.026116667  1.035553333  1.041156667  1.042126667  1.04673  1.049546667  1.05054  1.05532  1.065746667  1.083363333  1.089803333  1.09587  1.11665  1.12085  1.15371  1.181383333  1.20036  1.21962  1.220763333  1.22674  1.245843333  1.252893333  1.268896667  1.282893333  1.309863333  1.32082  1.335876667  1.336643333  1.353156667  1.354096667  1.456153333  1.458066667  1.473463333  1.549726667  1.603536667  1.618076667  1.619666667  1.62395  1.692906667  1.86127  2.060416667 | 3.1857E-05  0.002095263  0.00302709  0.006131013  0.003575894  6.73787E-05  1.84279E-05  1.75079E-05  2.08154E-05  0.006559312  0.014663719  3.70792E-05  4.54767E-05  0.0014959  0.001368346  7.60638E-05  0.082893723  0.00035528  0.002373401  0.00118369  0.005891979  0.00143335  1.94219E-05  0.129312291  0.000509838  0.017348109  0.002521638  0.000585028  0.002100427  0.370915539  1.46314E-05  4.73908E-05  0.0046468  6.59316E-06  0.008421419  0.009484608  0.000128434  0.031630848  1.82311E-05  0.011750982  8.84382E-05  0.000467995  0.000368333  0.0032358  0.012304258  0.000410205  0.008051196  0.012568815  0.001672443  0.00463862  0.001329985  0.006428302  0.04413068  0.005806318  0.000190214  0.009929897  0.005955457  0.007303  0.002053419  0.006660508  0.00030535  0.003080066  0.005141996  0.135289015  0.00070043  0.099367236  0.002951752  4.09227E-05  0.001142651  0.000459531  0.013764153  0.00125627  0.008780185  0.002044298  0.001129099  0.011020896  0.005419922  0.000480759  1.15592E-05  0.002280542  0.000240883  0.003285837  0.006973161  3.07147E-05  0.034554359  0.001319597  0.000835762  0.000179293  0.001782394  0.024040005  0.001194953  0.028093165  0.000752923  0.001043991  0.00128278  9.46059E-05  0.00117366  0.000189719  1.56899E-05  0.001871561  0.000120155  0.003934387  0.000808206  0.00847563  0.000118256  0.002599749  0.000159162  0.013477659  0.001641445  0.003519809  0.000481246  0.003872879  0.001199277  0.030223378  0.014602025  0.006689051  0.006759827  0.001334231  0.000534357  0.000855033  0.012009035  0.002656468  0.004080405  0.009874744  0.000446303  0.002356615  0.049015552  0.045755426  0.000793261  0.00397146  0.000132159  0.082399503  5.70649E-05  0.065300932  0.003070958  0.01625876  0.007611623  0.134788804  0.000629976  0.000584802  0.000379796  0.000269835  0.001206853  0.001069934  0.002270715  0.00635327  0.000405334  0.000462468  0.014037638  3.09931E-05  0.001366984  0.023658218  0.031473602  0.037238616  0.026253523  0.000424211  0.004792091  1.07957E-05  0.111140164  0.001780083  0.002855956  0.011489928  0.004168048  0.000401002  0.004209323  1.18803E-06  0.00039054  0.000239205  0.000716835  0.003814902  0.000397743  0.003986544  0.001426683  8.08086E-05  0.035829239  0.013278053  0.000491881  0.002169889  0.157370754  0.000806303  0.000310486  0.002506195  0.00021864  0.003896427  0.000529003  0.000518071  0.017314463  0.000247377  0.001243246  0.000146433  0.002825984  0.002997298  0.016060342  0.002389074  0.014856242  0.001717872  0.001735705  0.07953289  0.010302032  0.016875864  0.000391437  0.019260771  0.000329668  0.000277002  0.000200001  0.009011302  0.00481448  0.000862723  0.000677854  0.00643064  9.37568E-05  0.001254756  0.002707073  0.061596148  0.000240598  0.035535861  0.000807633  0.000740727  0.052591349  0.003764947  0.004343713  0.022909167  0.027510314  0.067005209  0.003293256  8.17998E-05  0.006139618  0.006911702  0.000342982  0.009728834  0.00048741  0.000256085  0.000139312  9.01857E-05  0.006423135  0.001275576  0.026044206  0.023011387  0.054114444  0.000416821  0.048725485  0.016208529  0.000822641  0.003501903  0.000726443  0.072293725  0.000746117  0.003553813  0.078802637  0.017338077  0.001782502  0.004747005  0.001118002  0.000252064  0.015656004  0.001288582  0.007593706  0.000267657  0.00080302  0.001124945  0.00126931  0.000239424  0.000127066  0.001047633  0.004372401  0.001075995  0.021804184  0.003450569  0.002011814  0.036710693  0.024856489  0.000556493  0.000842705  0.003765751  0.001556618  9.5102E-05  0.006876143  0.012800523  0.053406618  0.000399126  0.001018141  0.065932905  0.002349359  0.009812497  0.000650551  0.000744323  0.001805724  0.006412554  0.151189501  0.002141973  0.0008439  0.003946441  3.02405E-05  0.016203279  0.004520232  0.003312458  0.009794501  0.018373414  0.01214387  0.002417841  0.037985903  0.021263055  0.001085149  0.012629286  0.003936366  0.003812734  0.000729156  0.014949306  0.000198666  0.001075925  0.014782432  0.003036896  0.00635069  0.010563967  0.003207112  0.019178604  0.000976165  0.000561045  0.214235279  7.15371E-05  0.009018345  0.01429773  0.035598975  0.000535715  0.0005723  0.001828003  0.009134857  0.013671927  0.003853423  0.002352184  0.004017477  0.000305401  0.033824872  0.001892132  0.004520616  0.014722036  0.000336738  2.88635E-05  0.006618566  0.032453766  0.001941233  0.00100988  0.038158671  0.110178547  0.029443682  0.000554225  0.00053551  0.019996928  0.029804347  0.030583305  0.000770749  0.000841967  0.001286577  0.007390629  0.00074559  0.004836035  0.000168941  0.010405269  0.007272829  0.014302145  0.000467349  0.000387617  0.018999068  0.037659389  0.000446966  0.001407203  0.041538294  0.002887921  0.014785642  0.000874429  0.000838572  9.682E-05  0.1552143  0.00037294  0.002358325  0.190663882  0.001019948  0.000601029  0.003801118  0.002475101  0.001154736  0.008694643  0.001403218  0.000969424  0.000305468  0.000675282  0.001826137  0.000130239  0.012514824  0.06446016  0.000803128  0.001872392  0.002443118  0.012091492  0.002016803  0.003028758  0.000814817  0.003472905  0.0074833  0.014799128  0.040112557  0.00061402  0.010680398  0.003396615  0.005133817  0.074492141  0.010985576  0.001525812  0.002450118  0.000846426  0.003910688  0.004994374  0.075044337  0.001800606  0.010595771  0.097085748  0.00035147  0.000403902  0.012783184  0.00068326  0.00030267  9.54219E-05  0.000613734  0.045024042  0.02232707  0.000562642  0.012229208  0.016732448  0.002064098  0.001810103  0.001657561  0.000228534  0.009756254  0.00025666  0.001968273  0.000988483  0.001172071  0.000154648  0.03166078  0.005657757  0.016043326  0.008924135  0.062777228  0.004131558  0.0018459  0.000706826  0.040423641  0.101466107  0.020776286  0.001654001  0.039134738  0.002264409  5.04279E-06  0.033707134  0.001717348  0.001550987  0.019311099  0.009180065  0.000883761  0.020507841  0.050248833  0.001368895  0.001319931  0.013780577  0.000695274  0.00085904  0.004183717  0.004674186  0.001055369  0.000630924  0.090111449  0.008400885  0.007663067  0.004841987  0.000482585  0.001888398  0.001568759  0.000435151  0.000147326  0.003361279  0.00227492  0.000973826  0.015369815  0.013015242  0.031977221  0.053649571  0.007008043  0.078860201  0.000605989  0.000918724  0.105071313  0.002128297  0.010011686  0.000231568  0.001875589  0.021414927  0.01065137  0.000869051  0.037011399  0.011424743  0.002944686  0.007408982  0.063648845  0.001155622  0.024648642  0.001514689  0.00566324  0.0352418  7.78893E-05  0.009771059  0.007029326  0.001104224  0.023483781  0.002337259  0.000635556  0.000137834  0.017463294  0.058226555  0.084603307  0.00113829  0.001135399  9.95308E-05  0.018596841  0.02327956  9.3023E-05  0.049404096  0.095046694  0.000317596  0.000818947  0.005932401  0.073402048  0.008461759  0.005714453  0.020809179  0.021342106  0.009813285  0.00258363  0.008061821  0.068028646  0.000736596  0.011614005  0.002417232  0.004082742  0.000132362  0.004038697  0.003527912  0.017897053  0.007627865  0.051195836  0.000201019  0.00278064  0.000455229  0.000408065  0.026646471  0.009982661  0.000260744  0.055246021  0.008509461  0.001677578  0.038070301  0.019175633  0.00808845  0.03345365  0.000170076  0.008458116  0.001539898  0.004351036  0.002434726  0.00607096  0.004686695  0.017276609  0.002009002  4.32635E-05  1.77888E-05  0.022978862  0.000299183  0.000412184  0.001393969  0.01702514  0.092017905  0.010938502  1.60734E-05  0.002615274  0.019666072  0.005424709  0.01575024  0.004058337  0.000330556  0.003235658  0.002722199  1.61316E-05  0.029986825  0.005092224  0.002170292  0.003595882  0.000576236  0.036899772  0.000697914  0.001602264  0.000628893  0.001558042  0.006209822  0.000948072  0.000353165  0.005371994  0.000129965  0.007824389  0.004071223  5.64976E-05  0.000784808  0.019731165  0.000250828  5.35465E-05  0.000136461  0.005791905  0.00153107  0.018531171  0.026421697  0.000526763  0.00273828  0.067717158  0.020781233  0.000462514  0.001452706  0.000164075  0.006589397  0.004757629  0.011822726  0.008624874  0.177548386  0.004661552  0.004096833  1.94241E-05  0.000493348  0.011082366  0.00046567  0.001786216  0.001513046  0.135922668  0.000962883  0.007523045  0.024780638  0.00162035  4.52678E-05  0.000966966  3.27037E-05  0.02227252  0.000137141  0.000150047  0.000290749  0.007323964  0.003148307  0.046803245  0.002181141  0.002016171  0.007838777  0.002880515  0.000227097  0.000925469  0.005652885  9.96826E-05  0.060482645  0.000589647  0.094089908  5.36067E-05  0.003147192  0.001379939  0.020462623  0.006501569  0.003985199  0.001959305  0.000325054  0.002910484  0.004637379  0.090400772  0.00011553  0.000287299  0.000262651  0.095590494  0.014844405  0.02897277  0.002352694  0.006943118  0.011739048  0.00946255  0.004509656  0.003621091  0.002578613  0.000619041  0.024966833  0.000866003  0.001369495  7.82879E-05  0.018412867  0.000665395  0.005305121  1.1092E-05  1.00397E-05  0.004500534  0.002462429  3.60444E-05  0.002083706  0.000628582  0.005006492  0.051197204  0.006400937  0.207516855  0.001861353  0.001807879  0.008511126  0.014099777  0.000377936  0.000424836  0.09679967  0.001433676  0.000609005  0.019923334  0.000189858  0.004710324  0.000668703  0.002348587  0.003183469  0.00073761  0.006155708  0.000865711  0.011158446  0.017849192  0.000532876  0.005399477  0.003717103  0.014824505  0.004897763  0.00100407  0.000438031  0.000214208  0.001575194  0.033794572  0.000101834  0.005806686  0.000222645  0.009846823  0.001128181  0.00236281  4.86311E-05  0.002146224  0.57487686  0.000170385  0.021604816  0.010695655  0.006544768  0.002246916  0.009585316  0.004711688  0.000141394  0.000620994  5.10519E-05  1.97337E-05  3.366E-05  0.003576816  0.001153224  0.000847882  0.000954312  0.000312342  0.11947617  0.000830188  0.002420559  0.003288109  0.003187475  0.000314025  0.001143261  0.003989109  0.00042379  0.000869166  0.000315017  0.003203276  0.0039325  0.000528691  0.00883814  0.007585451  0.000594504  0.028746377  0.000524439  0.003148204  0.000806714  0.002817154  0.002079689  0.037389129  0.026895742  0.003059696  0.003647411  0.039054097  0.002889595  0.027848543  0.002007237  0.000151499  0.007469376  0.001715626  0.012031883  0.001522654  0.003937244  0.010972145  0.001668695  0.01930848  0.003454534  0.00377527  0.000323482  0.007032489  0.010586514  0.002823386  0.00841593  0.002089191  0.001104054  0.005345918  0.016326776  0.005833265  0.003162899  0.029337548  0.001955616  0.001131171  0.005656956  0.007020108  0.005049251  0.191623747  0.001628872  0.004182888  0.011810438  0.000461591  0.003082674  0.000305052  0.008139626  0.00334442  6.43712E-05  6.94887E-05  0.003635825  0.04735125  0.016020282  0.004075417  0.000733788  0.009408026  0.007639797  0.000121493  0.001673311  8.9917E-05  0.000278979  0.000321684  0.004349396  0.000193538  0.000243173  0.002029457  0.000135201  0.000114746  0.000117433  0.000219993  0.001070785  0.002092152  0.001680776  0.000986901  0.005312661  0.000457128  0.015377406  0.080670339  0.006271863  5.20378E-06  1.7183E-05  7.99595E-05  0.004639768  0.194235188  0.000142558  0.001206688  0.000606411  0.01393801  0.000243465  0.001942205  0.00524443  0.008275534  0.000212216  0.000137735  0.002915871  0.048307116  0.003305873  0.000863306  0.000168734  0.001742159  0.000208831  0.002609812  0.013510456  0.000207933  0.003554694  0.001107812  0.007815397  0.00146376  3.12366E-05  0.000586336  0.114633803  0.016137478  0.001707401  0.002134297  0.001031631  0.007607084  0.033051514  0.000990354  0.028548842  0.001606389  0.012413382  0.005614601  0.027348074  0.004186453  0.001840739  0.0068528  0.001875062  0.002096826  0.000449991  0.008872194  0.000395104  0.000873964  0.007229637  0.004161269  0.02973006  0.000310556  0.003517292  0.001147799  0.002038409  0.000499523  0.000275311  0.007296371  0.005603431  0.009967997  0.000937533  0.023856199  0.000149862  0.06542852  0.000330284  0.000897347  0.001768083  0.005649479  0.015031429  0.005880054  0.00396268  0.000774083  0.002891214  0.001877628  0.001406177  0.000634364  0.000856945  0.001775622  0.018121737  0.000339257  0.006128533  0.00636115  0.010677235  0.002557213  0.003826149  0.000253873  0.002360116  0.002566383  0.018599868  0.000905812  0.002171045  4.46852E-05  0.000953957  0.002661771  0.000471651  0.000733582  0.000444801  0.324119414  9.25068E-05  0.000783385  0.001280376  0.004153484  0.002583336  0.000536189  0.000176841  1.25225E-05  0.020583342  0.003078076  0.002991616  0.001925612  0.000324577  0.005458565  0.000529156  0.000286917  0.002610256  0.006796946  0.00103703  0.002136715  0.000195365  0.083496472  0.282141555  0.000678645  0.015657805  0.000369705  0.000102631  0.001980146  7.17341E-05  0.002892445  0.000541313  0.00065961  0.00465951  9.90336E-06  0.000176282  0.000103678  0.001523278  0.000419116  0.001079598  0.021016262  0.000205807  0.002833955  0.004157758  0.002455928  0.001198457  0.00106582  0.001384783  0.000443014  2.95328E-05  0.009288094  0.030720972  0.000159127  8.00708E-05  2.46308E-05  0.002427065  0.000440472  3.43335E-05  0.000499784  0.003650411  0.000212094  2.36682E-05  0.008087386  0.01662898  0.010378947  0.000507523  0.002480305  7.6701E-05  0.005067048  0.003333651  0.004513031  0.001458538  0.000488897  0.004083518  0.000817178  0.001668763  0.000200865  0.002868695  0.003111979  0.000787503  0.081376201  0.001152161  0.021131041  0.001153099  0.002605031  0.000538132  0.000714266  0.000964485  0.006481887  0.001904663  0.004367656  0.000607715  5.67366E-06  7.67177E-05  0.000405139  0.001000224  0.003465034  0.000438832  0.00592145  0.000399321  3.32825E-05  9.17267E-05  0.000102395  0.00142118  0.001893356  0.00016141  0.000676717  0.000493268  0.000290848 |

**
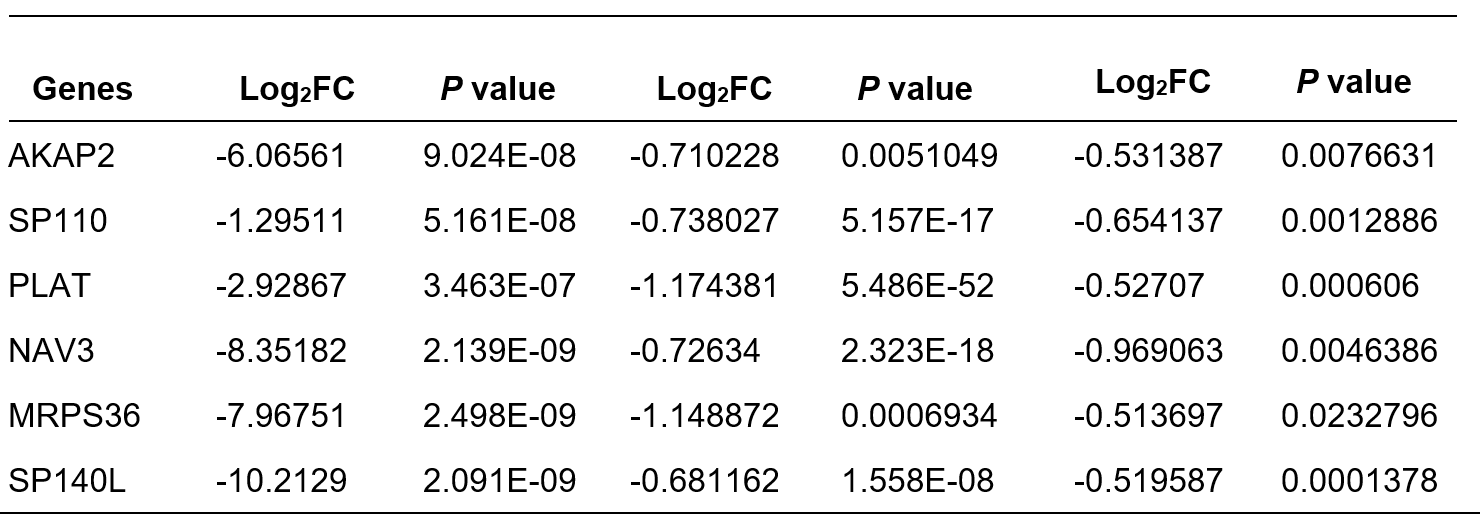
Table S4. A comprehensive comparison of the six convergent candidates**

m^5^C level

RNA level

Protein level

1. Zuo, S., et al., *NSUN2-mediated m(5) C RNA methylation dictates retinoblastoma progression through promoting PFAS mRNA stability and expression.* Clin Transl Med, 2023. **13**(5): p. e1273.

2. Yu, J., et al., *Nuclear PD-L1 promotes EGR1-mediated angiogenesis and accelerates tumorigenesis.* Cell Discov, 2023. **9**(1): p. 33.

3. Gu, X., et al., *Histone lactylation-boosted ALKBH3 potentiates tumor progression and diminished promyelocytic leukemia protein nuclear condensates by m1A demethylation of SP100A.* Nucleic Acids Res, 2024. **52**(5): p. 2273-2289.
